# Multistate and functional protein design using RoseTTAFold sequence space diffusion

**DOI:** 10.1038/s41587-024-02395-w

**Published:** 2024-09-25

**Authors:** Sidney Lyayuga Lisanza, Jacob Merle Gershon, Samuel W. K. Tipps, Jeremiah Nelson Sims, Lucas Arnoldt, Samuel J. Hendel, Miriam K. Simma, Ge Liu, Muna Yase, Hongwei Wu, Claire D. Tharp, Xinting Li, Alex Kang, Evans Brackenbrough, Asim K. Bera, Stacey Gerben, Bruce J. Wittmann, Andrew C. McShan, David Baker

**Affiliations:** 1 Department of Biochemistry, University of Washington, Seattle, WA USA; 2 Institute for Protein Design, University of Washington, Seattle, WA USA; 3 Graduate Program in Biological Physics, Structure and Design, University of Washington, Seattle, WA USA; 4 Department of Molecular Engineering, University of Washington, Seattle, WA USA; 5 Molecular & Cellular Biology, Medical Scientist Training Program, University of Washington, Seattle, WA USA; 6 Faculty of Engineering Sciences, Heidelberg University, Heidelberg, Germany; 7 School of Chemistry and Biochemistry, Georgia Institute of Technology, Atlanta, GA USA; 8 Office of the Chief Scientific Officer, Microsoft, Redmond, WA USA; 9 Howard Hughes Medical Institute, University of Washington, Seattle, WA USA

**Keywords:** Proteins, Computational models, Molecular engineering

## Abstract

Protein denoising diffusion probabilistic models are used for the de novo generation of protein backbones but are limited in their ability to guide generation of proteins with sequence-specific attributes and functional properties. To overcome this limitation, we developed ProteinGenerator (PG), a sequence space diffusion model based on RoseTTAFold that simultaneously generates protein sequences and structures. Beginning from a noised sequence representation, PG generates sequence and structure pairs by iterative denoising, guided by desired sequence and structural protein attributes. We designed thermostable proteins with varying amino acid compositions and internal sequence repeats and cage bioactive peptides, such as melittin. By averaging sequence logits between diffusion trajectories with distinct structural constraints, we designed multistate parent–child protein triples in which the same sequence folds to different supersecondary structures when intact in the parent versus split into two child domains. PG design trajectories can be guided by experimental sequence–activity data, providing a general approach for integrated computational and experimental optimization of protein function.

## Abstract (web summary)

ProteinGenerator simultaneously generates protein sequences and structures using sequence space diffusion.

## Main

Protein function arises from a complex interplay of sequence and structural features; hence, designing new protein functions requires reasoning over both sequence and structure space. Many protein design methods sample structures and sequences in separate steps, typically by generating protein backbones first and using inverse folding methods to generate sequences. Traditional methods, such as Rosetta flexible backbone protein design^1^, alternate between structure and sequence design, whereas recent deep-learning-based approaches^2–5^ typically generate backbones first and then use sequence design methods, such as ProteinMPNN (MPNN), to identify sequences that fold into a given backbone^6,7^. Among the latter class of approaches, denoising diffusion probabilistic models^8^ (DDPMs), which have shown considerable promise in continuous data domains, allow for the generation of protein backbones subject to a wide range of structural constraints^9^. DDPMs approximate the probability density function over a data distribution by learning to denoise samples corrupted with Gaussian noise, enabling the generation of high-quality samples from a Gaussian prior; they have been explored less in categorical domains, such as text and protein sequences^10^. Although powerful, structure-based approaches, such as RFdiffusion^11^ and Chroma^12^, provide limited opportunities to guide protein generation using sequence-based features and to identify sequences with more than one fold and/or function. Hallucination approaches that apply activation maximization to structure prediction networks^13,14^ can generate sequence–structure pairs without additional training, but these solutions can be adversarial and require a large number of steps to converge, and robust experimental success requires subsequent sequence design on the hallucinated backbones^7^.

**Fig. 1:**
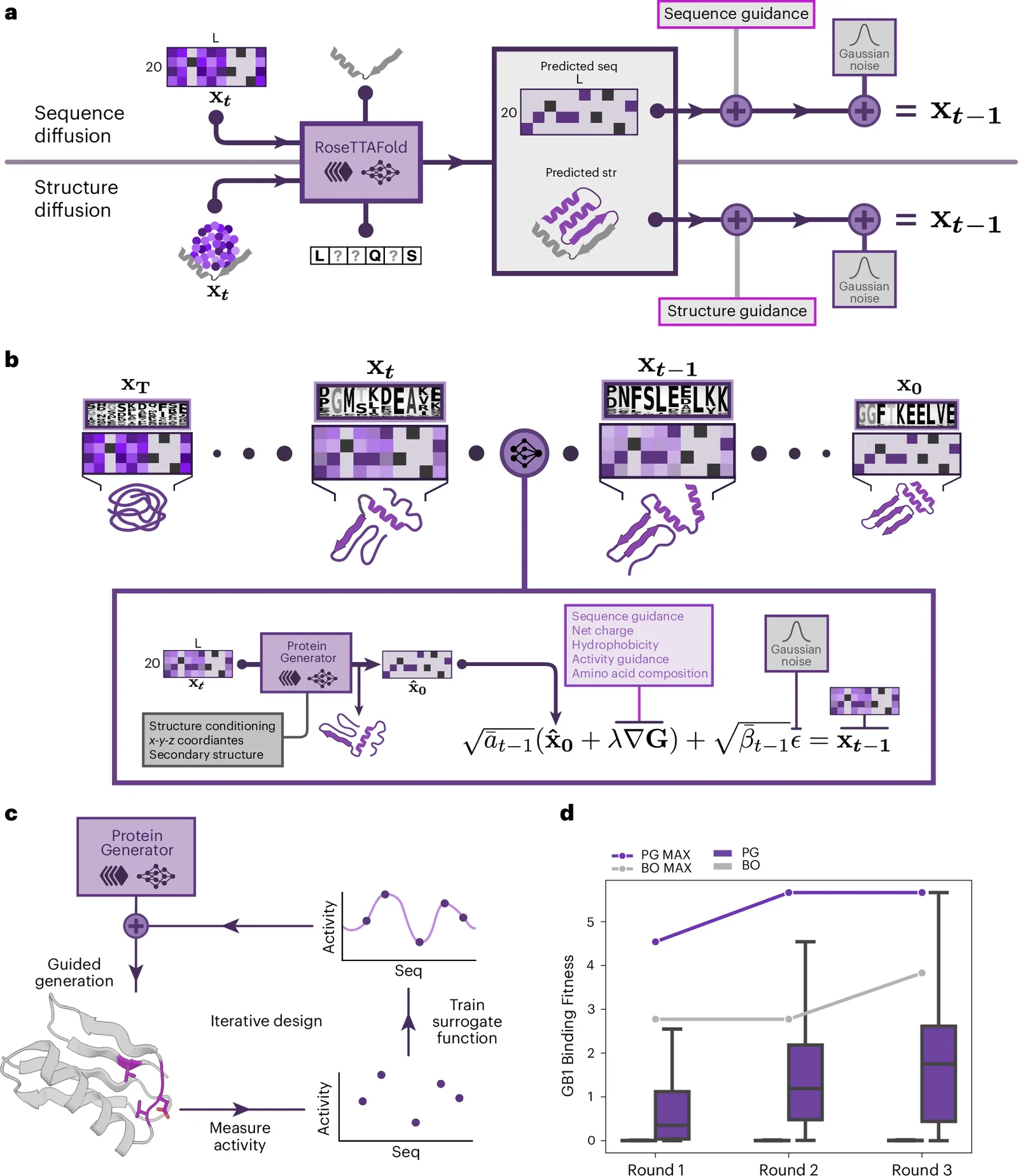
Overview of PG. **a**, Comparison of diffusion in sequence and structure space. PG and RFdiffusion take as input noised sequence (PG) or structure (RFdiffusion) data and problem specific sequence and structure constraints. At each denoising step, the RoseTTAFold architecture generates complete protein sequences and structures, and this is used to generate the next step in the trajectory in sequence (PG) or structure (RFdiffusion) space. Although specific structural or sequence features can be fixed in the input to RoseTTAFold in both approaches, biases toward particular sequence features during the diffusion update at each step are more readily incorporated in PG (as are biases toward structural features, such as symmetry, in RFDiffusion). **b**, Schematic of PG inference trajectory. At each step in the diffusion process the sequence **x**_0_ is predicted from sequence **x**_*t*_ by RF conditioned on any desired structural information, combined with any desired sequence bias, and noised to generate the **x**_*t*−1_. This process is repeated for T steps as the sequence–structure pair converges on a high-confidence solution shaped by the structural and sequence guidance information. **c**, Iterative design schematic demonstrating how PG can be used in an experimental feedback loop. Designs generated by the model are evaluated for activity; a surrogate function approximating sequence to function relationships is fit; and gradients from the surrogate can then be used to guide PG toward active design space. **d**, In silico demonstration of iterative design using GB1 fitness landscape for binding and comparison with Bayesian optimization (BO). In round 0, not shown in the plot, 96 designs are generated with PG without guidance, and a surrogate function is trained to discriminate high and low activity designs. In rounds 1–3, gradient-based guidance is used to generate 96 designs for each method; a surrogate function is fit; and the process is repeated. Line plots show maximum activity sampled, and box plots show distribution sampled over the batch of 96. Mean activities for each round are statistically significant between the two populations (*P* < 0.05, two-sided Mann–Whitney *U*-test, *n* = 96 designs per round). Box plots boundaries indicate upper and lower quartiles, and whiskers indicate the nearest quartile + 1.5× interquartile range. seq, sequence; str, structure.

We reasoned that carrying out diffusion in sequence space rather than structure space could enable guidance of design using sequence-based features and explicit design of sequences populating multiple states. To enable conditioning on both sequence and structure features, we start from the RoseTTAFold^15^ structure prediction network, which we treat as a mapping from input sequence and structure information to an output sequence and structure, as in the case of RFdiffusion (Fig. 1a and Supplementary Fig. 1). We reasoned that RoseTTAFold could be adapted for sequence space diffusion by noising the sequences of proteins in the Protein Data Bank (PDB; http://www.rcsb.org/) and training to remove the noise while imposing a loss on structure prediction accuracy, thus ensuring that the resulting model has a deep understanding of both sequence and structure.

## Results

### Categorical DDPM implementation and fine-tuning

We implement diffusion and data noising in categorical space by representing protein sequences as scaled one-hot tensors (for native sequences, true values are set to 1 and all other values set to −1) and embed via a linear layer, allowing for progressive corruption with Gaussian noise N(μ = 0, σ = 1)^16,17^. This approach has the advantage over carrying out diffusion in a learned embedding space^10,18^ of simplifying the use of raw sequence-based classifiers for guidance. To fine-tune RoseTTAFold, we input protein sequences progressively noised according to a square root schedule^10^, the corresponding timestep and optional structural information and train the model to generate ground truth sequence–structure pairs by applying a categorical cross-entropy loss to the predicted sequence (relative to the ground truth sequence) and FAPE^19^ structure loss on the predicted structure (Algorithm S1 and Supplementary Fig. 2). Self-conditioning^16^ was found to improve training and inference performance. Protein generation begins with an L×20 dimensional sequence of Gaussian noise and a black-hole^19^ initialized structure; at each timestep (**x**_*t*_), the model predicts **x**_0_ from **x**_*t*_, after which **x**_0_ is noised to **x**_*t*−1_ (Fig. 1b). Sequence-based guidance can be combined with **x**_0_ to guide the model toward a constrained sequence space using activity data, sequence-specific potentials or other information (Fig. 1b)^20^. Fixed motifs in the input sequence are featurized with an extra token to denote that the sequence is not diffused at this position. Secondary structure conditioning information is passed via the one-dimensional (1D) track, whereas three-dimensional (3D) coordinates are embedded via the pair features in the two-dimensional (2D) track and coordinates in the 3D track. Embeddings from these three tracks are linked by cross-attention in the RoseTTAfold architecture, allowing for the output sequence from the 1D track to condition the others.

During inference, we obtain **x**_0_ from **x**_*t*_ and generate **x**_*t*−1_ by noising **x**_0_; we found sampling **x**_*t*−1_ ~q(**x**_*t*−1_ | **x**_**0**_)^10^ more effective than sampling from **x**_*t*−1_ ~q(**x**_*t*−1_ | **x**_0_, **x**_*t*_)^8^ (Supplementary Fig. 3 and Algorithm S2). ProteinGenerator (PG) outperforms early hallucination methods in unconditional design accuracy and generates structurally diverse proteins when sampled from different Gaussian mixture models (Supplementary Figs. 4–6). PG readily designs proteins that scaffold specified structural motifs; AlphaFold2 (ref. ^19^) (AF2)-predicted structures accurately recapitulate (root mean square deviation (RMSD) to design < 2, motif RMSD < 1, AF2 pAE < 5) both the motif and the full design (Fig. 4c and Supplementary Fig. 8). RFdiffusion followed by MPNN performs better on motif scaffolding and unconditional generation of larger proteins (Supplementary Fig. 5c). PG sequence quality as measured by ESM^21^ pseudo-perplexity, which was previously shown to be indicative of experimental success^22,23^, is indistinguishable from those of native sequences sampled from UniProt^24^ and considerably higher than sequences generated using a 640-million-parameter sequence diffusion model, EvoDiff^25^ (Supplementary Fig. 5d,e).

Unconditional generation using PG yields sequence–structure pairs with amino acid compositions resembling those of native proteins (Supplementary Fig. 7). Structure predictions from AF2 and ESMFold^21^ of the generated sequences are close to the designed structures and confident (6% of designs have AF2 confidence pLDDT > 90 and RMSD < 2 Å; Supplementary Figs. 3 and 5; computational success rates for all design tasks can be found in Supplementary Table 1). We experimentally characterized unconditionally generated 70–80-residue proteins by testing solubility and monomericity via size-exclusion chromatography (SEC), folding by circular dichroism (CD) and stability by CD thermal melts (we use these methods throughout this study to evaluate protein behavior as the design criteria increase in complexity). Of the 42 proteins experimentally tested, 32 were soluble and monomeric by SEC, and CD experiments showed that they had the designed secondary structure and were stable up to 95 °C (Supplementary Fig. 9).

### Design of rare amino acid enriched proteins

**Fig. 2:**
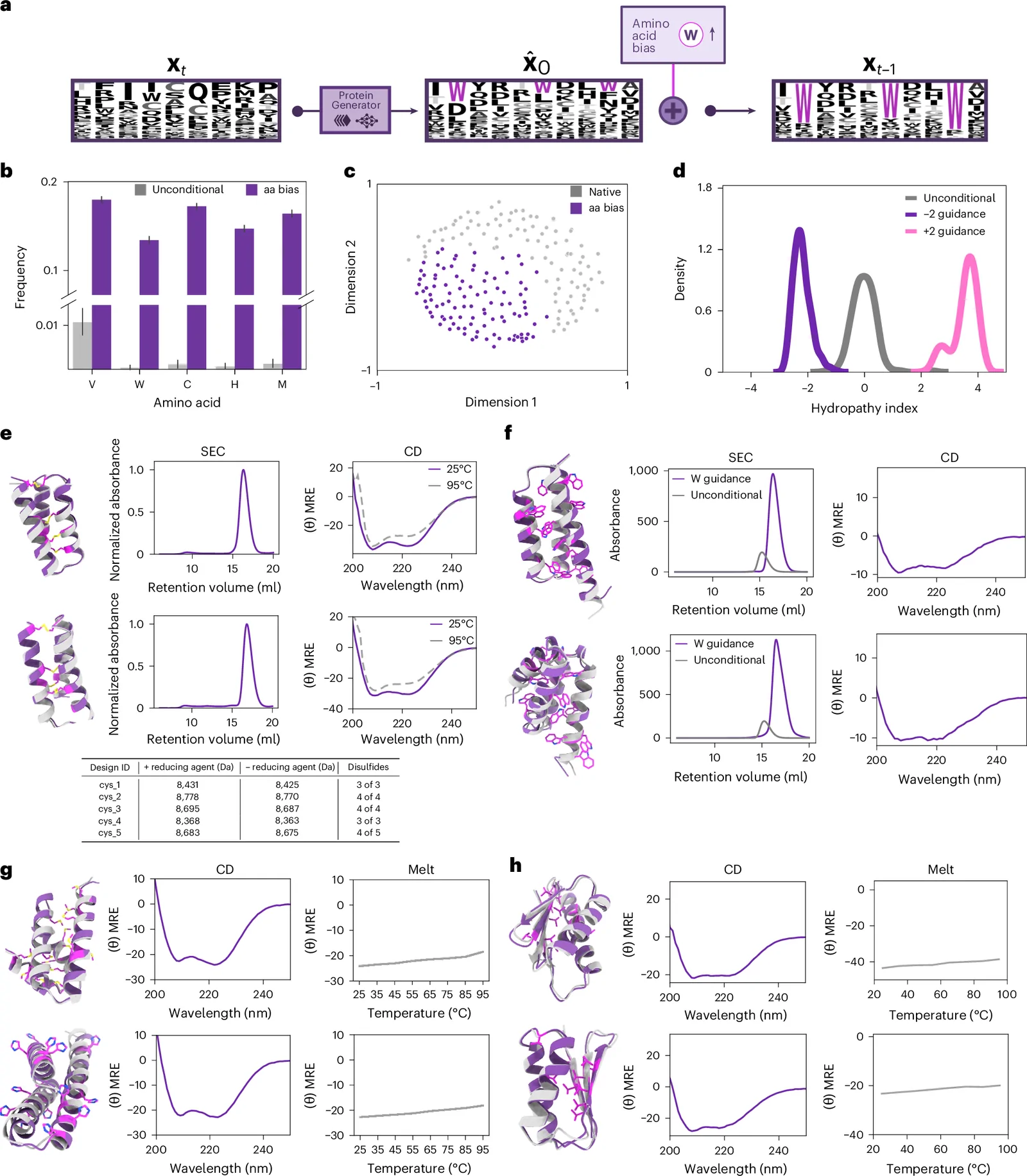
Design of proteins with specified sequence composition. **a**, Amino acid compositional bias schematic. **b**, Comparison of amino acid frequency in unconditional (gray) and amino acid biased (purple) generation; separate PG trajectories were carried out for each enriched amino acid. Error bars are standard deviation. Biased distributions are significantly different from unconditional amino acid frequencies (*P* < 0.05, two-sided Mann–Whitney *U*-test, *n* = 200 designs per amino acid). Box plot boundaries indicate upper and lower quartiles; whiskers indicate the nearest quartile + 1.5× interquartile range; and the center line is the median. **c**, Multidimensional scaling of native and amino acid biased sequences shows that they occupy distinct regions of sequence space. **d**, Hydropathy guidance. Biasing the sequence toward or away from hydrophobic amino acids results in a shifted distribution of hydropathy scores compared to unconditional generation (*P* < 0.05 two-sided Mann–Whitney U-test, *n* = 122 designs per condition). **e**, Experimental validation of cysteine biased designs (design in gray, AF2 in purple). Proteins are monomeric by SEC and alpha helical by CD at 25 °C and 95 °C. Mass spectrometry indicates the presence of the designed number of disulfide bonds. **f**, Experimental validation of tryptophan biased designs (design in gray, AF2 in purple). Designs are monomeric by SEC, have considerably higher absorbance at 280 nm than unconditional designs and are alpha helical by CD. **g**, Experimental validation of histidine and methionine biased designs (design in gray, AF2 in purple). **h**, Experimental validation of valine biased designs (design in gray, AF2 in purple). Valines highlighted in pink on the designs are present in the beta-fold secondary structure. CD traces and melt curves at 222 nm are to the right of the designs. CD traces and melt curves at 222 nm are to the right of the designs. aa, amino acid.

An advantage of diffusion in sequence space is that sequence-based guiding functions can be readily implemented and applied. To evaluate the ability of PG to reason over sequence–structure relationships outside the PDB training distribution, we sought to design proteins enriched in evolutionarily undersampled amino acids that confer structural or functional properties (Fig. 2a). Given a specification of the desired amino acid content, at each denoising step, sequence positions are ranked based on the frequency of the amino acid of interest, and, for the top N positions (where N is the number of desired occurrences of the amino acid), a bias toward the desired amino acid is added to the update generating **x**_*t*−1_ (Algorithm S3). We used this procedure to generate proteins with high frequencies (20% composition) of tryptophan, cysteine, valine, histidine and methionine (Fig. 2b and Supplementary Fig. 10a–e) with sequences very distinct from those of native proteins (Fig. 2c). Generated designs were filtered for high AF2 confidence (pLDDT > 90) and self-consistency (RMSD to design < 2 Å), and 96 were selected for experimental characterization.

Of the expressed designs, 68 were soluble in *Escherichia coli*, and four of five upweighted cysteine proteins, eight of 19 upweighted tryptophan proteins, 19 of 22 upweighted valine proteins, 10 of 12 upweighted histidine proteins and 10 of 10 upweighted methionine proteins were found to be monomeric by SEC (Supplementary Fig. 10g). CD spectra were obtained for a subset of the monomeric designs, and, in all cases, the indicated secondary structure was consistent with the design and thermostable (Fig. 2e–h). Guiding for high cysteine content at the sequence level resulted in the formation of 3–4 disulfide bonds per protein without any structural conditioning, as indicated by mass spectrometry in the presence and absence of the reducing agent TCEP at 50 mM (Fig. 2e and Supplementary Figs. 11–15). Proteins designed with upweighted tryptophans exhibited high absorbance at 280 nm and had helical CD traces (Fig. 2f). Proteins with upweighted valine exhibited higher beta-sheet content (Supplementary Fig. 10f) by CD, as expected given the secondary structure propensity of valine^26^, and were thermostable (Fig. 2h). These results indicate that the model can reason over sequence–structure relationships beyond native protein-like sequence compositions to design folded, thermostable proteins with desired sequence properties.

We further explored the generation of proteins with pre-specified charge composition, isoelectric points and hydrophobicity^27^. We implemented sequence-based potentials to guide^20^ the diffusive process toward these characteristics to enable fine-tuned control over physical properties of the output sequence. This approach enabled the design of proteins with a range of user-defined hydrophobicities (Fig. 2c) and isoelectric points (Supplementary Fig. 10h). The ability of PG to control sequence properties during backbone generation should be useful for increasing the developability of therapeutic candidates^28^.

### Design of sequence repeat proteins

**Fig. 3:**
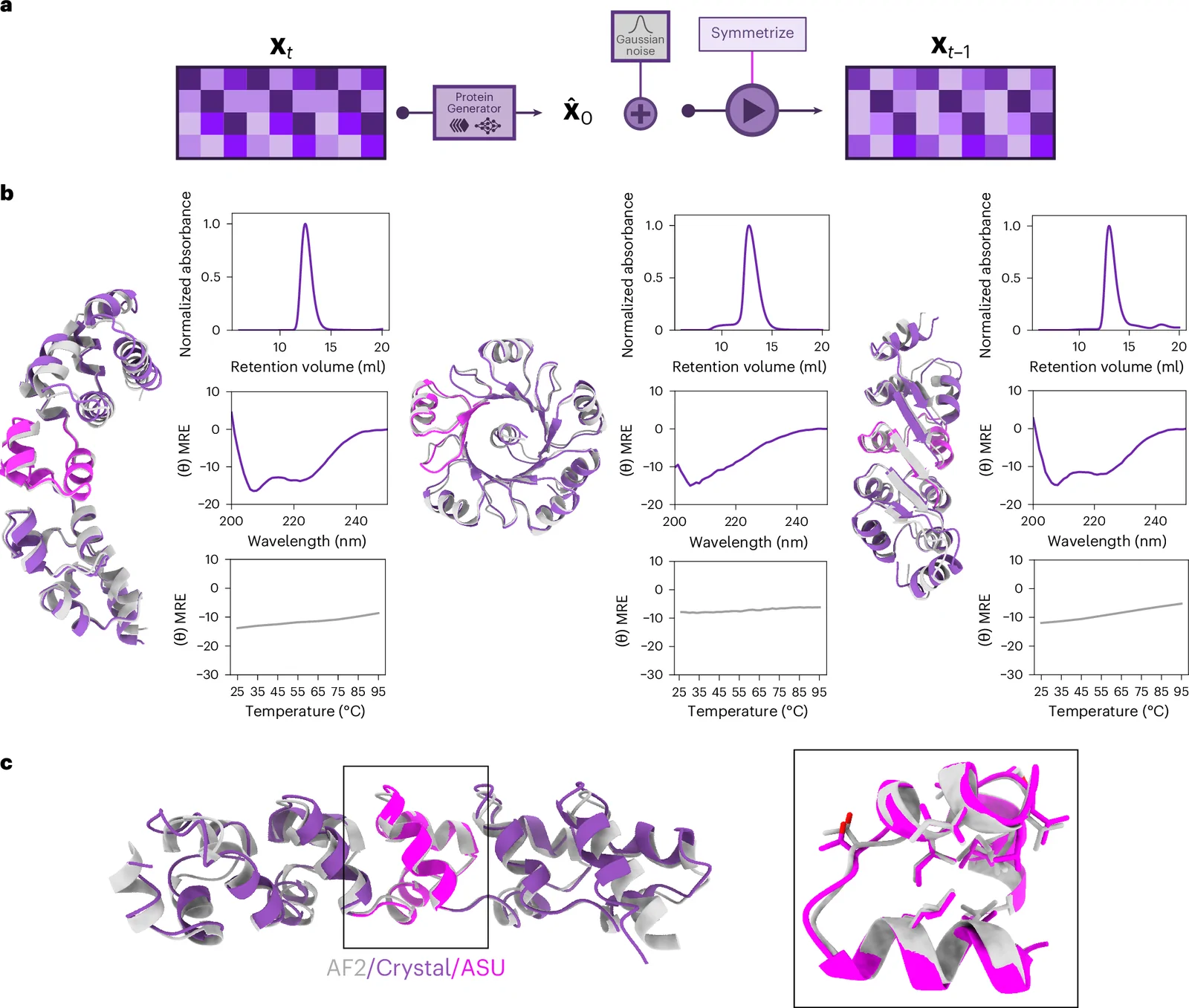
Design of sequence repeat proteins with PG. **a**, Symmetric sequence diffusion to design proteins with sequence symmetry. **b**, Experimental validation of sequence repeat proteins. Designs in gray are overlaid with AF2 predictions in purple, and asymmetric units are highlighted in pink. SEC and CD traces and melting curves demonstrate stability of these designs. **c**, 3.70-Å crystal structure of designed repeat protein: AF2 model in gray, crystal structure in purple and asymmetric unit in pink. Box on the right highlights the accuracy of designed side chains in the asymmetric unit.

Repeat proteins containing tandem copies of a sequence–structure unit are ubiquitous in nature and play central roles in molecular recognition and signaling^29^. Previous repeat protein design work has required pre-specification of structural features^30^ or expensive Markov chain Monte Carlo (MCMC) calculations^7^. We reasoned that PG could be adapted readily to generate repeat proteins given only the sequence length of the repeat unit and number of repeats desired by, at each timestep, applying repeat symmetry to the noised sequence distribution (Fig. 3a). Unconditional generation with this approach yielded largely beta-solenoid structures. To encourage further exploration, we trained PG to condition on secondary structure (Supplementary Fig. 16) and specified secondary structure constraints to yield a wide range of all-alpha, all-beta and mixed alpha/beta designs (Fig. 3b). We added helical caps^31^ to a subset of designs to promote stability and reduce aggregation^32,33^. We experimentally characterized 74 repeat proteins with helical caps and 86 repeat proteins without helical caps. Of these, 27 repeats with caps and 10 repeats without helical caps were soluble and monomeric by SEC, and seven of eight proteins evaluated using circular dichroism had the expected secondary structure (Fig. 3b and Supplementary Fig. 17). We solved the crystal structure of a five-repeat unit design composed of a four-helix bundle asymmetric unit and found the design to have atomic accuracy: the C RMSD of design to the crystal structure was 1.38 Å for the whole structure and 0.47 Å for the asymmetric unit (Fig. 3c and Table 1).

**Table 1:** Data collection and refinement statistics for the repeat protein crystal structure

	CAP repeat (PDB: 8VD6)
Resolution range	72.08–3.70 (4.05–3.70)
Space group	*P2*1
Unit cell	21.62, 45.05, 72.11
	90.00, 91.55, 90.00
Unique reflections	1,543 (359)
Multiplicity	2.0 (2.0)
Completeness (%)	99.8 (99.7)
Mean I/sigma(I)	11.6 (7.6)
Wilson B-factor	76.23
R-merge	0.047 (0.093)
CC1/2	0.997 (0.974)
Reflections used in refinement	1,534 (359)
Reflections used for R-free	79 (79)
R-work	0.2465 (0.2465)
R-free	0.2847 (0.2847)
Number of non-hydrogen atoms	1,384
Macromolecules	1,384
Protein residues	174
RMS (bonds)	0.002
RMS (angles)	0.59
Ramachandran favored (%)	88.95
Ramachandran allowed (%)	7.56
Ramachandran outliers (%)	3.49
Average B-factor	62.38
macromolecules	62.387

Statistics for the highest-resolution shell are shown in parentheses.

### Design of conditionally active peptide cages for membrane lysis

**Fig. 4:**
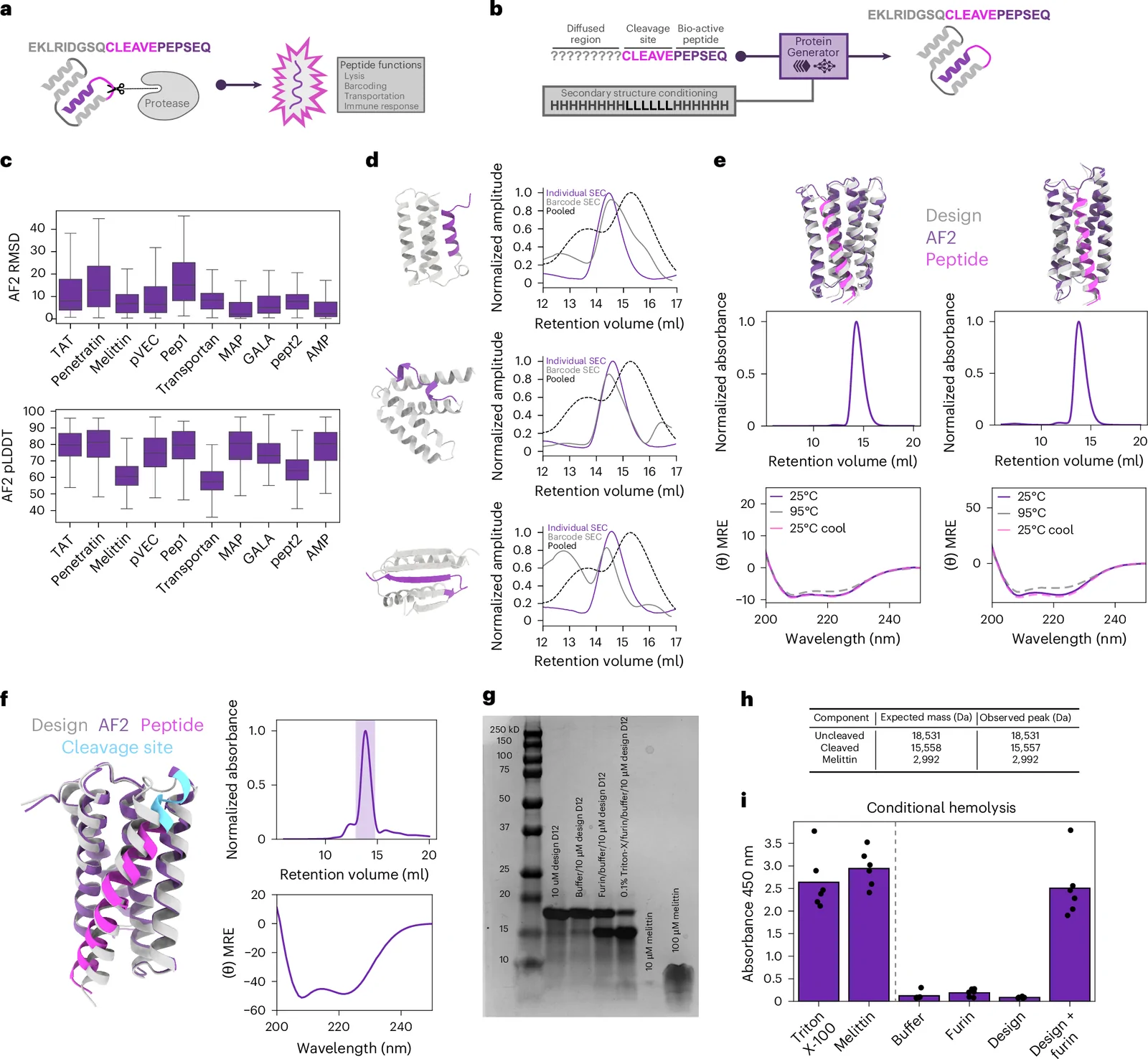
Scaffolding bioactive peptides and intrinsic barcodes with PG. **a**, Schematic overview of functional peptide scaffolding for downstream tasks such as protease cleavage for lysis and peptide barcoding. **b**, Sequence-only motif scaffolding and secondary structure conditioning to generate proteins with embedded functional sequences. Cleavage sites can be specified at the N or C terminus of the peptide to allow for protease cleavage. **c**, In silico design metrics for sequence-only bioactive peptide scaffolding. RMSD of AF2 predictions to designs on the top and AF2 pLDDT of designs on the bottom. Box plot boundaries indicate upper and lower quartiles; whiskers indicate the nearest quartile + 1.5× interquartile range; and the center line is the median. *n* = 2,000 designs per condition. **d**, Mass spec peptide barcoding assay. Scaffolding barcodes with PG results in soluble and monomeric designs by SEC. SEC traces for individual designs are in gray. When the same designs are expressed in a pooled library (black), and fractions are digested with trypsin, analytical mass spectroscopy of each fraction is able to recapitulate the SEC trace shown in purple. **e**, Melittin scaffolded designs with furin cleavage site. Designs are shown in gray, and AF2-predicted structures are shown in purple, with melittin peptide highlighted in pink. Designs are soluble and monomeric by SEC and folded with helical secondary structure by CD. **f**, Melittin scaffolded design D12. D12 design model is in gray; AF2-predicted structure is overlayed in purple for scaffold; cyan is for the cleavage site; and pink is for melittin. SEC fraction of monomeric D12 used for downstream assays is highlighted with the purple bar. CD trace of D12 is consistent with the designed helical secondary structure. **g**, Representative SDS-PAGE of uncleaved D12 (18 kD), cleaved D12 (15 kD) and melittin peptide (3 kD) (*n* = *3* biological replicates). **h**, Mass spec of the cleavage reaction products confirms the presence of uncleaved D12, cleaved D12 and melittin. Melittin mass was calculated with an additional c-terminal ‘GS’ due to the expression vector used. **i**, Absorbance at 450 nm for six technical replicates of washed RBCs after incubation with design with and without furin protease. Positive controls Triton X-100 and melittin are shown to the left of the vertical bar. Design with furin lyses RBCs significantly more than samples without design (*P* = 0.002, two-sided Mann–Whitney *U*-test) or furin (*P* = 0.005, two-sided Mann–Whitney *U*-test) and is on par with positive controls Triton X-100 (*P* = 0.127, two-sided Mann–Whitney *U*-test) and melittin (*P* = 0.132, two-sided Mann–Whitney *U*-test). Source data

The design of proteins with activities conditional on an external input is of considerable interest for the design of therapeutics^34^ and biosensors^35^ with spatial and temporal control. We used PG to address this challenge by scaffolding bioactive peptide sequences within an inert protein cage (Fig. 4a) by designating a region of the protein chain (usually at the N or C terminus) to be fixed at the bioactive peptide sequence and freely diffusing the remainder of the sequence. Unlike the previous LOCKR^36,37^ sensor system, in which the bioactive sequence must be in a helical conformation and make specific interactions with the caging scaffold, neither the structure the peptide adopts nor the structure of the scaffold needs to be pre-specified, enabling caging of a much broader range of peptide sequences (Fig. 4b). Given the peptide sequence and scaffold length, PG generates designs that contain the peptide sequence as an integral part of the protein structure and are predicted to fold with greater than 85 pLDDT and less than 2-Å RMSD to the designed scaffold (Fig. 4c). We used this approach to design proteins caging the pore-forming peptide melittin that can be conditionally released upon proteolytic cleavage of a terminal loop. We specified the sequence of the bioactive peptide melittin with an adjacent furin cleavage site and used secondary structure conditioning to scaffold the peptide in a helical bundle with the cleavage site in a loop to improve protease access (Fig. 4b). Because of the multiple constraints (scaffolding the melittin sequence in an ordered structure and a furin cleavage sequence in a loop), this required increased sampling and filtering (Supplementary Table 1). Despite melittin being disordered in isolation, PG was able to generate solutions with the melittin sequence adopting a helical structure, which we then experimentally tested. Of 13 experimentally characterized designs, five were soluble and monodisperse by SEC, folded with helical secondary structure by CD and thermostable (Fig. 4e,f). We extended the cleavage loop and inserted interface arginine mutations to promote disassociation of the peptide after cleavage (Fig. 4f). Upon addition of furin protease, a band shift from −18 kD to −15 kD indicated cleavage of our designed melittin cage (Fig. 4g). Mass spectrometry analysis confirmed release of intact melittin peptide (Fig. 4h and Supplementary Fig. 18). To test conditional membrane lysis of our caged melittin protein, we incubated red blood cells (RBCs) with design D12 in the presence or absence of furin protease and measured absorbance at 450 nm to quantify the presence of heme from lysed RBCs. Samples pre-incubated with furin protease were bright red in color, indicating membrane lysis, whereas little lysis was observed without furin before treatment (Fig. 4l and Supplementary Fig. 19). Due to the bioavailability of endogenous endosomal proteases, such as furin, we anticipate that the design of caged peptides will enable a route toward endosomal escape.

### Scaffolding barcode peptide sequences

Peptide barcoding enables large libraries of proteins to be screened in binding assays or SEC, with the identity of individual proteins subsequently read out by mass spectrometry of the barcode after release by proteolysis^38^. Because of the challenge of incorporating a barcode within a folded protein, current approaches attach peptide barcodes to proteins of interest through N-terminal or C-terminal flexible fusions, but these can affect both expression and solubility, and, hence, it can require some 5–10 degenerate barcodes to infer the behavior of the untagged protein. Given the promising results of our bioactive peptide scaffolding experiments, we reasoned that PG could scaffold short barcode-like sequences (7–14 residues) into a protein of interest, thereby removing the need for multiple extrinsic barcodes. We scaffolded a set of validated peptide barcodes^7,39,40^ at the C-terminus, flanked by lysine and arginine on the N-termini and C-termini, respectively, to permit facile tandem cleavage with Lys-C and trypsin. PG generates designs that contain the peptide sequence as an integral part of the protein structure and are predicted to fold with reasonable AF2 confidence and RMSD to the design (Fig. 4c and Supplementary Fig. 20). We cloned, expressed and purified a pilot library of 84 pooled designs and used SEC to separate them by size. Barcodes were isolated from each fraction and run on an Orbitrap Lumos to determine the identities of the proteins in each fraction, as described previously^7,39,40^. As a control, we expressed and purified each of these 84 proteins and subjected them to SEC; of these, 64 of 84 (76%) were expressed, and 48 of 64 (75%) of the expressed proteins exhibited monodisperse elution peaks at the expected elution volume (Supplementary Fig. 21a). We overlaid individual SEC (iSEC) elution profiles of expressing designs with corresponding reconstructed iSEC barcode traces (73/84) normalized to maximum MS2 intensity (SEC-MS) for a set of 58 designs. We observed close agreement between SEC-MS and iSEC peak elution volume for 41 of 58 (71%) designs (Supplementary Fig. 21b); exemplary traces are shown in Fig. 4d and Supplementary Fig. 22. Thus, PG can incorporate intrinsic barcodes into protein libraries, resulting in shorter constructs than required with external barcoding approaches.

### Multistate design

Designing an amino acid sequence that can adopt distinct structural conformations upon an external trigger is a challenging task, as the energy landscape must contain two discrete minima with free energy differences small enough for a trigger to induce state switching^41^. We reasoned that PG was well equipped for explicit multistate design because guidance can be applied from multiple condition-dependent structural constraints during the sequence diffusion process. To adapt PG to multistate design, we input to RoseTTAFold the same sequence but different structural conditioning information and take a linear combination of the output logits as input to the next timestep.

**Fig. 5:**
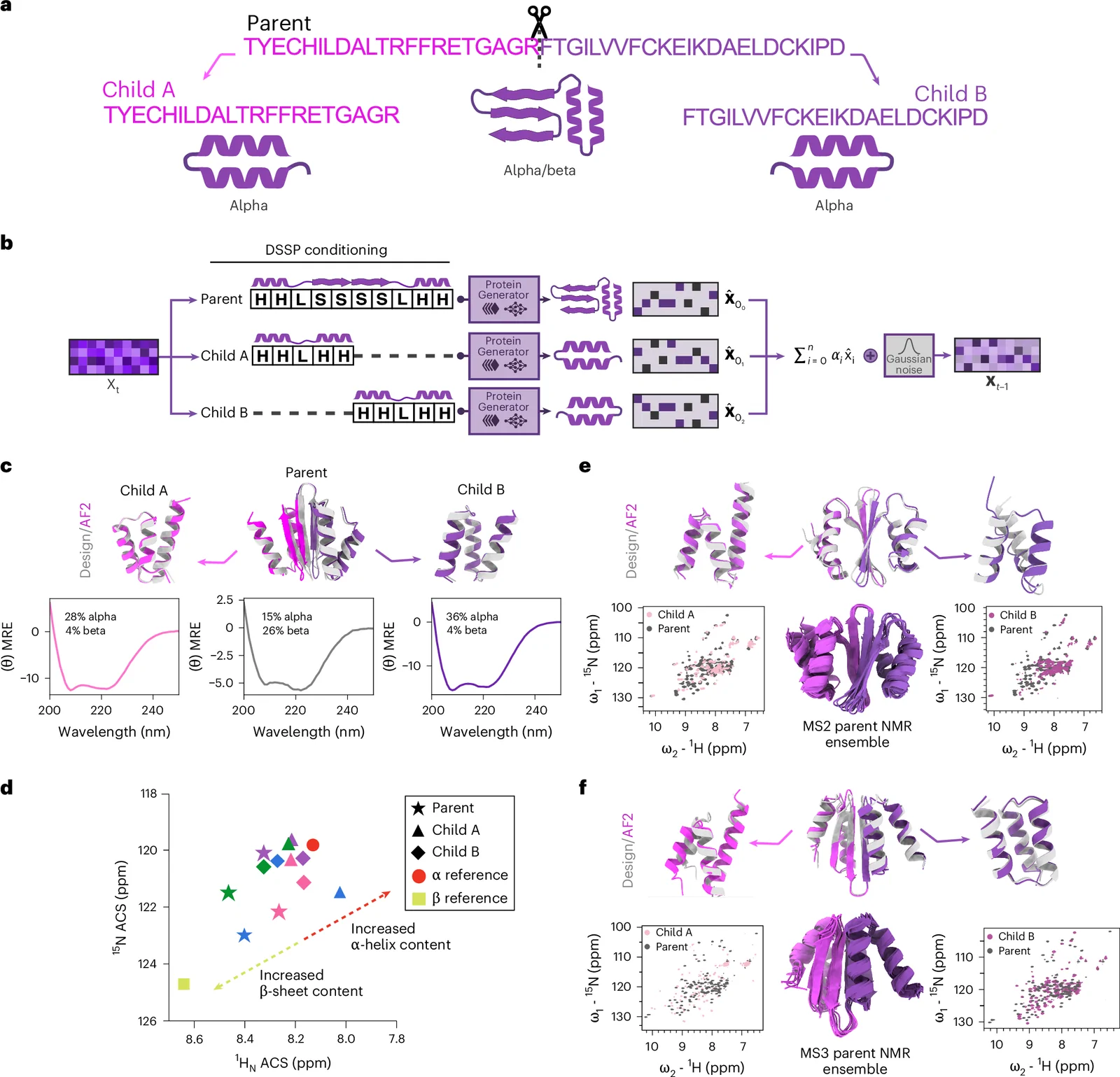
Multistate design with PG. **a**, Multistate DSSP conditioning is used to generate a sequence with an alpha/beta fold in the parent state and all alpha in the child A and child B states. **b**, Implementation of multistate DSSP sequence conditioning. Different DSSP conditioning strings are applied to a full-length parent sequence and two subsequences (child A and child B). RoseTTAFold predictions and model logits are output for parent, child A and child B. A linear combination of output logits is used as a potential to guide the model toward finding one sequence that satisfies all DSSP conditioning strings for parent, child A and child B. **c**, MS1 family adopts distinct folds by CD. Top, high pLDDT design and AF2 models of family MS1. Bottom, CD spectra and deconvolution of family MS1 indicating 26% beta content in the parent compared to 4% beta content in child A and child B, respectively. **d**, ACS of ^1^H_N_ and ^15^N chemical shifts values obtained from MS1–MS4 HSQC spectra. Reference average ACS values of primarily α-helical proteins (red circle) and primarily β-sheet proteins (yellow square) are shown calculated from ^1^H_N_–^15^N correlations using chemical shift information obtained from the Biological Magnetic Resonance Bank. ACS values are compared for multistate sequences among parent (α/β mix fold), child A (α-helical fold) and child B (α-helical fold). MS1 in pink, MS2 in purple, MS3 in blue, MS4 in green. MS2 (**e**) and MS3 (**f**) families are designed by PG to adopt distinct folds in the parent and child states with high AF2 confidence (top row). HSQC overlays of MS2 and MS3 child A and B compared to parent (bottom row; ω indicates chemical shift). NMR structures of MS2 and MS3 parent fold into the intended secondary structures with atomic-level accuracy (bottom middle).

We used this approach to design protein sequences that adopt distinct folds when connected in a single chain (the parent) or when expressed separately or cleaved into two chains by a protease (child A and child B) (Fig. 5a). At each timestep, **x**_*t*_, we use RoseTTAFold to model the full-length parent sequence along with the cleavage products child A and child B and average the resulting logits followed by noising to **x**_*t*−1_ (Fig. 5b and Algorithm S4). DSSP^42^ features are appended to the L×20 sequence representation of each family member to enable conditioning on protein secondary structure (Fig. 5b). We used this approach to generate multistate sequences (MS) that are designed to adopt specific ɑ/β folds in the parent state and different all ɑ-helical folds in the child states; as expected given the greater problem complexity, this required more sampling than the above single sequence design problems (Supplementary Table 1).

We experimentally characterized 72 parent–child triples that AF2 predicted with high confidence and accuracy to be in the parent state when intact and the child states when split (Supplementary Fig. 23), and we selected 4 (MS1–MS4) soluble and monodisperse sequence families for detailed CD and nuclear magnetic resonance (NMR) studies (Supplementary Fig. 24a–d). 2D ^1^H-^15^N amide heteronuclear single quantum coherence (HSQC) spectra revealed that all the MS1–MS4 parents and children were well folded and globular proteins (Supplementary Fig. 24). CD spectra of all of the children were consistent with all-alpha proteins; spectral deconvolution suggested higher beta-sheet content in the parents (Fig. 5c and Supplementary Fig. 24, middle rows). As secondary structure estimation by CD can be imperfect, we took advantage of the fact that NMR chemical shifts are influenced by local secondary structure, which leads to distinct averaged chemical shift (ACS) values for primarily α-helical versus β-sheet proteins^43,44^. As expected for proteins with increased β-character, ^1^H and ^15^N ACS values of all parent designs were shifted downfield relative to the two associated children (Fig. 5d, dotted yellow arrow), which were shifted upfield into the reference region associated with α-helical proteins (Fig. 5d, dotted red arrow). Clear differences in chemical shift positions of MS1–MS3 child A and child B NMR peaks relative to the parent suggest that they adopt distinct folds (Fig. 5e,f; the differences were smaller for MS4 (Supplementary Fig. 24d), which the ACS values (Fig. 5d) and AF2 predictions (Supplementary Fig. 24e) suggest may not adopt a single fold). Taken together, these data suggest that, as intended, the designed child sequences fold into ɑ-helical supersecondary structures distinct from the parent designs.

To assess the accuracy of the designed alpha/beta parent folds, we obtained high-resolution structures of MS2 parent and MS3 parent (Fig. 5e,f and Table 2). The solution NMR structure of MS2 parent is within 1.06 Å Cα RMSD of the design model, with the central beta-sheet nearly perfectly recapitulated. The solution NMR structure of MS3 parent is within 1.61 Å Cα RMSD of the design model, with the beta-sheet again very close to the design model. These high-resolution structures of the parents, together with the all-alpha helical ACS values and CD spectra of the children, strongly suggest that, as designed, there are large-scale structural rearrangements upon splitting of the polypeptide chain.

**Table 2:** NMR structure statistics for designed multistate proteins

	MS3 parent (PDB: 8VL4)	MS2 parent (PDB: 8VL3)
Assignment completeness (backbone atoms N, HN, CA, CB, CO)	93%	94%
NMR distance and dihedral constraints		
Distance constraints		
Total NOEs	59	140
Intra-residue (|*i*–*j* | = 0)	0	0
Sequential (|*i*–*jj* | = 1)	43	65
Medium-range (|*i*–*j* | >1 and |*i*–*j* | <5)	9	63
Long-range (|*i*–*j* | ≥ 5)	7	12
Intermolecular	0	0
Hydrogen bonds	0	0
Total dihedral angle restraints	192	196
Total residual dipolar coupling (RDC) restraints	0	0
Total chemical shift restraints	464	468
Structure statistics		
Violations		
Distance constraints (total no.)	8	11
Dihedral angle constraints (°)	0	0
Max. dihedral angle violation (°)	0	0
Max. distance constraint violation (Å)	1.65	0.74
Deviations from idealized geometry		
Bond lengths (Å)	0	0
Bond angles (°)	0	0
Impropers (°)	0	0
Average pairwise backbone root means squared (r.m.s.) ensemble (Å)	1.02	1.16
Ramachandran analysis		
Favored	98 ± 1%	95 ± 1%
Allowed	2 ± 1%	5 ± 1%
Disallowed	0 ± 0%	0 ± 0%

### Guidance with experimental data

A longstanding goal in directed evolution is the optimization of desired functional attributes, such as enzyme activity, in as few experimental iterations as possible. We investigated the use of PG for experimental data-driven protein function optimization. As a test case, we used an experimental benchmark dataset on the IgG-binding protein GB1, which has the advantage of completeness: activity was measured for every sequence combination (for the four residues that were varied), allowing for evaluation of the activity of any sequence generated for these residues using PG. For other datasets where only a small fraction of sequences have known fitness, it is difficult to do such retrospective comparisons (because the PG-generated sequences will almost always have unknown fitness, like the vast majority of other possible sequences)^45^.

We simulated an iterative guidance process by exploring the fitness landscape of the protein GB1 via gradient-based optimization with IgG binding activity-guided diffusion trajectories. At each step, we biased sampling by gradients from classifiers trained on the experimentally determined fitnesses of preceding rounds (Fig. 1c). As the classifiers, we used two-layer multilayer perceptrons (MLPs) optimized for GB1 fitness greater than 2. For broad applicability, we carried out this optimization with standard settings and no extensive hyperparameter turning (96 designs generated and tested per round for three rounds). For comparison, we tested multiple optimization approaches using different acquisition functions on this same problem and chose the best performer to compare to PG. We found that the average and maximum fitness of PG-generated designs increases each round, outperforming a Bayesian optimization baseline with the best identified acquisition function (batched upper confidence bound) (Fig. 1d and Supplementary Figs. 25 and 26). The improved performance likely reflects the rich prior understanding of protein sequence–structure relationships implicit in RoseTTAFold compared to the baseline, which has access to only limited experimental data. Our PG approach can readily incorporate any experimentally measurable fitness attribute and should be useful for machine-learning-assisted directed evolution campaigns^46–48^.

## Discussion

The in silico and experimental results presented here demonstrate that PG can readily generate a wide variety of de novo (Supplementary Fig. 27) proteins subject to diverse sequence domain constraints, including amino acid composition bias, repeat sequence symmetry, bioactive peptide caging and multistate design. In this section, we compare PG to RFdiffusion and highlight the areas where PG sequence space diffusion is particularly advantageous. Both PG and RFdiffusion take advantage of RoseTTAFold to jointly model protein sequences and structures, and, hence, both PG sequence space diffusion trajectories and RFdiffusion structure space diffusion trajectories can be guided by both sequence and structure information. For example, given ‘hard constraints’, such as the identities of amino acids at certain positions, or the 3D structure of part of a protein (for an enzyme active site, typically both types of constraints would be provided), both methods will generate proteins satisfying the constraints by providing the relevant sequence and structure input at each RoseTTAFold denoising step along with the current partially denoised sequence (PG) or structure (RFdiffusion). However, ‘softer’ sequence constraints, such as biases on the number of amino acids of a given type, are more readily implemented in sequence space diffusion, whereas global structural properties, such as overall symmetry, are more readily implemented in structure-based diffusion. As with other protein generative models, obtaining sequences predicted to satisfy specific problem constraints required considerable filtering; future work will seek to increase the fraction of generated sequences that are confidently predicted.

Sequence space diffusion is specifically advantageous in multistate design of protein sequences that fold to two or more distinct structures as in our parent child designs; this can be implemented in sequence space diffusion by logit averaging, but it is not straightforward with structure space diffusion. PG enables deep-learning-based multistate design through the joint search of sequence and structure space without assuming fixed structural priors and should be readily generalizable to the design of more complex conditional state-switching protein systems; evaluation of these designs will benefit from advances in methods for structure ensemble prediction^49–51^. Beyond multistate design, we expect PG, and, more broadly, generative methods enabling direct sequence based guidance, to be useful in generating successive rounds of sequences for experimental characterization in directed evolution campaigns^46,52^. Although classifiers trained on the available experimental data can be used to directly generate sequences using Bayesian optimization and other approaches, using these classifiers instead to guide PG diffusion trajectories has the considerable advantage of being informed by the rich sequence–structure prior information represented within the PG network, which increases the likelihood that the generated sequences will fold and function.

## Methods

### Sequence representation

To apply the diffusion framework in sequence space, a continuous representation of the categorical sequence data is needed. To implement this, we represented the sequence, **x**_0_, with dimensions L×20 where L corresponds to the protein length with 20 possibilities for each amino acid type. This takes the form of a one-hot encoded vector that is centered at zero by multiplying the L×20 tensor by 2 and subtracting 1. Each logit within the tensor is a real number, with higher values corresponding to a higher probability for that specific amino acid at that position. With this representation, we noise **x**_0_ to obtain **x**_*t*_ with the below equation following the Ho et al.^8^ formulation for a standard forward process sampling from Gaussian noise with mean at 0 and standard deviation of 1.

$$q({{\bf{x}}}_{t}|{{\bf{x}}}_{0})={\mathscr{N}}({{\bf{x}}}_{t};\sqrt{{\bar{\alpha }}_{t}}{{\bf{x}}}_{0},(1-{\bar{\alpha }}_{t}){\bf{I}})$$

A critical part of the forward diffusion process is selecting the noising schedule. Determining the correct bin of a categorical distribution is trivial at low timesteps by argmaxing the input sequence. Therefore, more noise should be present at low timesteps to increase the difficulty of the task during training. The square root noise schedule^10^ satisfies this requirement and was employed in this study.

### Training

To train the model, we began by sampling *t* uniformly from [0,T], where *t* = 0 is an un-noised sequence and *t* = T is pure Gaussian noise. We then noise **x**_0_ to **x**_*t*_ with equation (1) and tasked the model to predict the un-noised sequence **x**_0_ and its corresponding structure **y**. The timestep feature was added to the sequence template passed to the model. We applied a categorical cross-entropy loss to **x**_0_ and structure losses to **y** (FAPE, bond angle, bond length, distogram, lddt). An additional KL loss^10^ was applied to the calculated **x**_*t*__−1_, as previously demonstrated to stabilize training of discrete diffusion models^10^. Self-conditioning^16^ was implemented to allow the model to condition on the previous **x**_0_ prediction and the back-calculated **x**_*t*−1_ during both training and inference. To self-condition in practice, the model was used with gradients turned off to first predict **x**_0_ from **x**_*t*+1_, which was then passed in as a sequence template to the model. During training, RoseTTAFold was allowed 1–3 uniformly sampled ‘recycle’ steps to refine structure predictions via multiple passes through the model^53^. Pseudo training and inference code is available in the Supplementary Information (Algorithms S1 and S2). In later training iterations, secondary structure conditioning was provided to the model by concatenating a tensor representing DSSP features onto the sequence template. These features were provided 25% of the time and masked uniformly between 0% and 90% when provided.

Along with the standard diffusion task (40% of the time), the model was also challenged with structure prediction (seq2str) and fixed backbone sequence design (30% of the time each). Incorporating these additional tasks during training helped maintain the agreement of sequence–structure pairs diffused by the model. Training examples were conditioned on sequence or structure by either unmasking 1–4 spans of residues, each 4–8 amino acids in length to simulate motif scaffolding, or unmasking randomly selected residues for the model to scaffold as an active site scaffolding problem. Unmasked structure conditioning information was supplied to the input for RoseTTAFold as templates in the 1D sequence track as well as the 2D and 3D structural information tracks.

### Inference

During inference starting from **x**_*t*_, the model predicts **x**_0_ and simultaneously decodes it to **y**. **x**_0_ is then back-calculated to **x**_*t*__−1_ with equation (1) and passed through the network with the previously predicted **x**_0_ to apply self-conditioning. Benchmarking against conditioning on **x**_*t*_, as done in Ho et al.^8^ with the below equation, shows that this approach performs better (Supplementary Fig. 3c), as seen in other categorical diffusion methods^10,17^.

$$q({{\bf{x}}}_{t-1}|{{\bf{x}}}_{t},{{\bf{x}}}_{0})={\mathscr{N}}({{\bf{x}}}_{t-1};{\tilde{{\boldsymbol{\mu }}}}_{t}({{\bf{x}}}_{t},{{\bf{x}}}_{0}),{\tilde{\beta }}_{t}{\bf{I}}),$$

where $${\tilde{{\boldsymbol{\mu }}}}_{t}({{\bf{x}}}_{t},{{\bf{x}}}_{0}):=\frac{\sqrt{{\bar{\alpha }}_{t-1}}{\beta }_{t}}{1-{\bar{\alpha }}_{t}}{{\bf{x}}}_{0}+\frac{\sqrt{{\alpha }_{t}}(1-{\bar{\alpha }}_{t-1})}{1-{\bar{\alpha }}_{t}}{{\bf{x}}}_{t}$$ and $${\tilde{\beta }}_{t}:=\frac{1-{\bar{\alpha }}_{t-1}}{1-{\bar{\alpha }}_{t}}{\beta }_{t}$$

This is done for T steps, but T can be varied and does not have to be what was used during training (inference time for fixed T can be found in Supplementary Table 2). The model finds solutions to some problems in as few as 10 steps (Fig. 1c). Furthermore, clamping the model’s output logits from −3,3 gives better agreement with AF2 predictions (Supplementary Fig. 3b). **x**_*t*−1_ is sampled from either a zero-mean normal distribution or a non-Bayesian Gaussian mixture distribution with equal mixing probabilities. For the non-Bayesian Gaussian mixture models, we defined a mixture with two normals centered at [−1, 1] (GMM2) and a mixture with three normals centered at [−1, 0, 1] (GMM3).

### Unconditional protein generation

Unconditionally generated proteins were assessed against a set of 1,000 native proteins with a length deviating up to five residues randomly sampled from the RCSB^54^ database. For experimental verification, proteins ranging from 70 to 80 amino acids in length with no conditioning information were generated in 25 steps. Designs were filtered by AF2 pLDDT > 90 and AF2 RMSD to design < 2 Å for ordering final constructs. Additionally, proteins with high model confidence but moderate AF2 confidence were ordered by filtering on design pLDDT > 90, AF2 pLDDT < 80 and AF2 RMSD to design < 5 Å.

### Compositionally biased protein generation

Proteins ranging from 70 to 80 amino acids in length with an amino acid compositional potential were generated in 25 steps. Designs were filtered by AF2 pLDDT > 90, AF2 RMSD to design < 2 Å and SAP score^55^ < 30. The top 10–22 designs were ordered for each upweighted amino acid type (tryptophan, cysteine, valine, histidine and methionine). Pseudocode for the implementation of the amino acid compositional potential is provided in the supplements (Algorithm S3).

### Charge biased protein generation

Proteins of 50 amino acids in length with charge potentials applied were generated in 25 steps with charge conditioning information. The ground truth charge for each protein was calculated at pH 7.4 by using the Henderson–Hasselbach equation.

### Hydrophobic biased protein generation

Proteins of 50 amino acids in length with hydrophobic potentials applied were generated in 25 steps with hydrophobicity conditioning information. The ground truth hydropathy index for each design was calculated by summing the hydropathy index for each residue and dividing by the sequence length^56^.

### DSSP guidance

For constructing the DSSP features, we calculated each training example’s DSSP based on the structure with helix, strand, loop and masked labels^57^. During training, the calculated per-residue secondary structure features were appended to RoseTTAFold’s 1D features and were one-hot encoded for 25% or 50% of the time and masked for 30% or 80% of the time. During inference, DSSP features are appended to the 1D features as necessary and masked when not. Secondary structure representations were input to the model as follows: H, helix; E, sheet; L, loop; X, masked.

### Repeat protein generation

Repeat proteins ranging from 125 to 150 amino acids in length were generated in 50 steps with and without DSSP conditioning information. Designed proteins contained five repeat units using one of the following DSSP strings, where X represents mask, E represents strand and H represents helix:‘XXXXEEEEEXXXXXXXXXXXXXXXHHHHHXXXX’, ‘XXXXEEEEEXXXXXHHHHHXXXXXEEEEEXXXX’, ‘XXXXHHHHHXXXXXEEEEEXXXXXHHHHHXXXX’, ‘XXXXHHHHHXXLXXHHHHHXXLXXHHHHHXXXX’, ‘XXXXEEEEEXXHXXEEEEEXXHXXEEEEEXXXX’, ‘XXXXXXXXXXXXXXXXXXXXXXXXXXXXXXXXX’, ‘XXXXEXXXXXXXXXEXXXXXXXXXEXXXXXXXX’. Designs were filtered on AF2 pLDDT > 80 and AF2 RMSD to design < 2 Å.

### Caging bioactive peptides

Proteins of 155 amino acids in length were generated in 25 steps with spans of helical DSSP conditioning to encourage the model to generate helical bundles and cleavage loops. Additional DSSP features were provided to scaffold the furin protease cleavage site ‘GRRKR’. The sequence for melittin was provided without DSSP conditioning as N-terminal 26 amino acids ‘GIGAVLKVLTTGLPALISWIKRKRQQ’. Designs were filtered by AF2 pLDDT > 85, AF2 RMSD to design < 2 Å and SAP scores < 40.

### Scaffolding barcode peptide sequences

Proteins of 100 amino acids in length were generated in 50 steps with barcodes being set as fixed regions on the C-terminus. Designs were filtered by AF2 pLDDT > 85 and AF2 RMSD to design < 2 Å.

### Multistate guidance

Parent and child protein pairings were generated in 25 steps using secondary structure features ‘XHHHHHHHHHHHHHLLLHHHHHHHHHHHHHLLLEEEEEELLLEEEEEXXXXXXXEEEEELLLE EEEEELLLHHHHHHHHHHHHHLLLHHHHHHHHHHHHHX’ for parent, ‘XHHHHHHHHHHHHHLLLHHHHHHHHHHHHHLLLHHHHHHHHHHHHHHHHHHHX’ for child A and ‘XHHHHHHHHHHHHHHLLLHHHHHHHHHHHHHLLLHHHHHHHHHHHHHX’ for child B. A mixing coefficient of 0.25 was used to combine parent and child sequences together at each step. Multistate design pseudocode implementation is available in the supplements (Algorithm S4).

### Partial noising

To promote further exploration of known active sequence subspaces, we implemented partial noising during design trajectories. This was done by introducing a temperature parameter, λ ∈ (0 to 1], to PG that, at the beginning of a trajectory, sets t to round (T*λ) rather than T (complete noising). This forced the model to do local exploration of the partially noisy sequence subpace. This approach was done in conjunction with normal design trajectories in ‘multistate guidance’ with λ parameters iterated through [0.2,0.3,0.5,0.8] starting with passing AF2 design sequences.

### Iterative guidance

Iterative guidance was employed as described in Fig. 1c. In short, the experimental or in silico characterization of designs is collected to train a classifier, which is used to generate further designs with guidance. In an iterative process, classifiers trained on designs sampled in a preceding round inform network sampling. We investigated the nearly complete fitness landscape of the V39, D40, G41 and V54 amino acid sites of GB1, which is the binding domain of protein G, an immunoglobulin binding protein found in streptococcal bacteria, as provided in the FLIP^58^ paper. Three rounds of iterative design were conducted with a batch size of 96.

We performed diffusion on the four mutation sites in the GB1 protein while providing guidance using a scale of 2.0 in the second-round and third-round design processes. In the first round, designs are directly sampled from our protein generator. For each round, a vanilla MLP (two layers, rectified linear unit (ReLU) activation and dropout of 0.25 after the first layer) is trained on the designs generated in the preceding round using a fitness equal to 1 as the classifier boundary. For comparison, batched Bayesian optimization was performed for three rounds using the sequences generated in the first round of the iterative guidance as the initial dataset. For Bayesian optimization, we used Gaussian processes with the radial basis function (RBF) kernel for the surrogate function, and we used the Monte Carlo–based batch upper confidence bound (qUCB) acquisition^59^ function with λ = 0.5.

### Sequence and structure quality metrics

All designs used in the ESMFold^21^ benchmarks were modeled by using the following curl command: [curl -X POST–data ‘SEQUENCE’] (https://api.esmatlas.com/foldSequence/v1/pdb/). All designs that were used in AF2 benchmarks and ordered for experimental characterization were predicted in single-sequence structure prediction mode with model 4. Pairwise backbone RMSDs between the design model and AF2 model were calculated for each design. Sequence quality metrics measured by ESM^21^ (pseudo) perplexity were calculated with model: esm2_t33_650M_UR50D.

### Sequence identity calculations

Blast alignment was used to examine sequence alignment and similarity using query coverage >90% and target coverage >50%. Alignment to natives was done against UniRef90 (Supplementary Fig. 27).

### Plasmid construction

Protein designs were cloned into plasmids as in Watson et al.^11^. In brief, designs were ordered as synthetic genes (eBlocks, Integrated DNA Technologies) with BsaI overhangs compatible with a ccdB-encoding expression vector, LM0627 (ref. ^7^). Genes cloned into LM0627 result in the following sequence: MSG-design-GSGSHHWGST**HHHHHH** (SNAC cleavage tag and **6×His affinity tag** are indicated). We used the NEBridge Golden Gate Assembly Kit (New England Biolabs) with a total reaction volume of 5 μl and a ratio of 1:2 by mass of LM0627 plasmid DNA to design. We then incubated the reaction mixture at 37 C for 30 min, halted the reaction by incubating the reaction mixture at 60 °C for 5 min and transformed 1 μl of the reaction mixture into 6 μl of BL21 competent cells (New England Biolabs). After heat shock and recovery in SOC media, transformed BL21 cells were grown overnight in 1.0 ml of LB from which glycerol stock was created and small-scale expression cultures were inoculated.

### 1-ml-scale protein purification

Initially, proteins were expressed with small-scale expression screens as previously reported^7^ with small adaptations. In brief, designs were inoculated with 100 µl of overnight growths and 900 µl of auto-induction media (sterile-filtered TBII media supplemented with 50 µg ml^−1^ kanamycin, 2 mM MgSO_4_, 1 × 5,052) in deep-well 96-well plates. Sixteen hours after inoculation, cells were harvested and lysed in lysis buffer (50 mM Tris-HCl (pH 8), 0.5 M NaCl, 30 mM imidazole supplemented with 1× BugBuster, 1 mM PMSF, 0.1 mg ml^−1^ lysozyme, 0.1 mg ml^−1^ DNase). Clarified lysates were added to a 50-μl bed of Ni-NTA agarose resin in a 96-well fritted plate equilibrated with wash buffer (50 mM Tris-HCl (pH 8), 0.5 M NaCl, 30 mM imidazole). After sample application and flowthrough, the resin was washed three times with wash buffer, and samples were eluted in 200 μl of elution buffer (50 mM Tris-HCl (pH 8), 0.3 M NaCl, 0.5 M imidazole, 5 mM EDTA (pH 8)). All eluates were sterile filtered with a 96-well 0.22-μm filter plate (Agilent, 203940-100) before SEC. Protein designs were then screened via SEC using an ÄKTA FPLC outfitted with an autosampler capable of running samples from a 96-well source plate. Samples were run on a Superdex S75 Increase 5/150 GL column (Cytiva, 29148722; 3,000–70,000-Da separation range) in a running buffer (20 mM Tris (pH 8), 150 mM NaCl). To improve peak resolution, the SEC column was connected directly in line from the autosampler to the UV detector. Then, 0.25-ml fractions were collected from each run. Absorption spectra were collected by an ÄKTA U9-M at 230 nm and 280 nm.

### 50-ml-scale protein purification

Proteins selected for further downstream characterization were expressed in 50 ml of auto-induction media^60^. Sixteen hours after inoculation, cells were harvested and lysed in lysis buffer (50 mM Tris-HCl (pH 8), 0.5 M NaCl, 30 mM imidazole, 1 mM PMSF, 0.1 mg ml^−1^ lysozyme, 0.1 mg ml^−1^ DNase) through sonication. Clarified lysates were added to a 2-ml bed of Ni-NTA agarose resin in a 20-ml column (Bio-Rad, 7321010) equilibrated with wash buffer (50 mM Tris-HCl (pH 8), 0.5 M NaCl, 30 mM imidazole). After sample application and flowthrough, the resin was washed three times with 10 ml of wash buffer, and samples were eluted in 2 ml of elution buffer (50 mM Tris-HCl (pH 8), 0.5 M NaCl, 200 mM imidazole). All eluates were sterile filtered with a 3-ml 0.22-µm filter plate before SEC. Protein designs were then screened via SEC using an ÄKTA FPLC outfitted with an autosampler capable of running samples from a 96-well source plate. Samples were run on a Superdex S75 Increase 10/300 GL column (Cytiva, 29148721; 3,000–70,000-Da separation range) in a running buffer (20 mM Tris (pH 8), 150 mM NaCl). Then, 1-ml fractions were collected from each run. Absorption spectra were collected by the ÄKTA U9-M at 230 nm and 280 nm.

### 0.5-L-scale protein purification and SNAC cleavage

The best expressing proteins used in high-resolution structural studies were selected for further scale-up and SNAC cleavage^61^. Proteins were expressed in Studiers M2 autoinduction media with 50 µg ml^−1^ kanamycin. Pre-cultures were grown overnight. Cultures were inoculated with 10 ml of pre-culture and grown at 37 °C for 4 h before lowering temperature to 22 °C for 14 h, and cultures were inoculated with 10 ml of pre-culture. Cells were pelleted at 4,000*g* for 10 min, after which the supernatant was discarded. Pellets were resuspended in 30 ml of lysis buffer (100 mM Tris HC (pH 8), 100 mM NaCl, 400 mM imidazole, 1 mM PMSF, 1 mM DNase). Cell suspensions were lysed by sonication for 7.5 min (10 s on, 10 s off) at 80% amplitude using a Qsonica four-prong sonicator. The lysate was clarified at 14,000*g* for 30 min. The His-tagged proteins were batch bound for 1 h to 8 ml of Ni-NTA resin (Qiagen) and washed with 10 ml of lysis buffer and 30 ml of high-salt wash buffer (25 mM Tris HCl (pH 8), 1 M NaCl, 40 mM imidazole) and then 10 ml of SNAC cleavage buffer (100 mM CHES, 100 mM acetone oxime, 100 mM NaCl, 500 mM GnCl (pH 8.6)). Next, 40 ml of SNAC cleavage buffer and 80 µl of 1 M NiCl_2_ were added, and columns were closed and shaken on a nutator for 12 h to cleave. After cleavage, the flowthrough was collected and concentrated before further purification by SEC/FPLC as described above.

### Cysteine bias protein expression

Proteins guided toward high cysteine content were transformed into and expressed in Rosetta-gami B(DE3) competent cells (Novagen, 71137). The 1-ml and 50-ml scale protein purification protocols were otherwise followed.

### CD

CD spectra were collected on a Jasco J-1500 CD spectrometer with 1-nm bandwidth, 50-nm permanent scan rate and data integration time of 4 s per read. Sample cuvettes stored in 2% Hellmanex (Hellma, 9-307-011-4-507) were washed with deionized water, 2% Hellmanex, deionized water and then 20% ethanol, after which 300 µl of SEC-purified protein was added for CD spectra measurements. Thermal melts were performed in 10° intervals between 25 °C and 95 °C.

### Mass spectrometry

To identify the molecular mass of each protein, intact mass spectra were obtained via reverse-phase liquid chromatography–mass spectrometry (LC–MS) on an Agilent G6230B TOF on an AdvanceBio RP-Desalting column and subsequently deconvoluted by way of BioConfirm using a total entropy algorithm. Disulfide formation was determined by injecting protein at 1.5 mg ml^−1^ in the presence and absence of 50 mM TCEP-HCl (Millipore Sigma, 646547-10X1ML) and detecting the mass shift.

### Disulfide bond quantification

To measure the number of cysteines via alkylation, proteins at 1.5 mg ml^−1^ in SEC running buffer (20 mM Tris (pH 8), 150 mM NaCl) were incubated in 50 mM TCEP-HCl at 50 °C for 1 h to reduce disulfide bonds. Simultaneously, an equal amount of protein in SEC running buffer was heated to 50 °C without 10 mM TCEP to maintain formed disulfides. Iodoacetamide (Millipore Sigma, I1149) was added to both conditions to a final concentration of 10 mM and incubated away from light at room temperature for 30 min to alkylate unpaired cysteines. To identify the molecular mass and alkylations status of each protein, intact mass spectra were obtained via reverse-phase LC–MS on the Agilent G6230B TOF on an AdvanceBio RP-Desalting column and subsequently deconvoluted by way of BioConfirm using a total entropy algorithm.

### Barcode extraction and liquid chromatography–tandem mass spectrometry

Ni-NTA eluate of the 84-design pool was subjected to SEC with deep fractionation (0.25-ml fractions). From every other fraction, 100 µl was added to fresh wells in a 96-well plate, and fractions were subjected to cleavage in 100 µl of Lys-C buffer (8 M urea, 100 mM Tris HCl (pH 8)) plus 1 µg of endoproteinase LysC (New England Biolabs, P8109S), as previously described. After hexaHis-tagged barcode pulldown with magnetic His-pulldown beads (Thermo Fisher Scientific, 10103D) and subsequent trypsin (New England Biolabs, P8101S) digest to free barcodes, barcodes were diluted 50% in 0.1% trifluoroacetic acid (TFA). Barcode pools corresponding to SEC fractions were separated by hydrophobicity using a previously described tandem guard column–analytical column setup. The guard column was packed to 2 cm with 5 µm of silica (ReproSil-Pur 120 C18Aq, ESI Source Solutions, r15.aq.0001), whereas the analytical column was packed to 14 cm with 1.9 µm of silica (ReproSil-Pur 120 C18Aq, ESI Source Solutions, r119.aq.0001). Peptides were detected using a previously described data independent acquisition (DIA) protocol on a Orbitrap Fusion Lumos Tribrid (Thermo Fisher Scientific) at the UW Proteomic Resource (UWPR).

### Solution NMR

Recombinant plasmid DNA (~100 ng) containing synthetic genes encoding for child A, child B and parent for several design families were separately transformed in *E. coli* BL21(DE3) cells. Colonies were grown under kanamycin selection on LB agar media for 16 h. Toward preparation of uniformly ^15^N-labeled proteins, a streak of colonies was resuspended in 60 ml of 1× M9 minimal media^62^ and grown overnight at 37 °C/225 r.p.m., and the inoculum was used to initiate a 1-L 1× minimal media culture supplemented with kanamycin and ^15^N ammonium chloride (Cambridge Isotope Laboratories, NLM-467) as the nitrogen source. For ^15^N/^13^C-labeled proteins, ^13^C-labeled glucose (Cambridge Isotope Laboratories, CLM-1396) was used as the carbon source. Cultures were incubated at 37 °C/225 r.p.m. until the optical density at 600 nm (OD_600_) reached 0.6 and then induced with 1 mM IPTG and grown at 37 °C/225 r.p.m. for 6 h. Cultures were harvested by centrifugation (6,000*g*, 15 min, 4 °C), and cell pellets were resuspended with wash buffer (300 mM NaCl, 10 mM imidazole, 50 mM Tris (pH 8)). Cells were lysed by sonication on ice. The lysate was clarified by centrifugation at 10,000*g* for 20 min at 4 °C. The supernatant was loaded onto a 5-ml His-Trap Ni-NTA column. The column was washed extensively with wash buffer, and protein was eluted using a linear gradient from 0% to 100% elution buffer (300 mM NaCl, 500 mM imidazole, 50 mM Tris (pH 8)). Fractions containing protein were pooled and further purified by SEC on a Superdex 200 Increase 10/300 GL column in NMR buffer (100 mM NaCl, 20 mM sodium phosphate (pH 6.5)). All designs were purified into batch-matched NMR buffer and then concentrated to 300 μl in 3-kDa Amicon concentrators. The purity of eluent fractions was confirmed to be greater than 95% by SDS-PAGE. Protein concentrations were measured by NanoDrop spectrophotometer at 280 nm with extinction coefficient predicted by ExPASY ProtParam. 2D ^1^H-^15^N amide HSQC spectra (Bruker pulse sequence hsqcetf3gpsi) were acquired using standard parameters at a ^1^H field of 800 MHz at 37 °C with recycle delay (d1) set to 1.2 s, sweep width of 30 ppm and acquisition time of 60 ms and number of scans ranging from 8 to 64 on a Bruker AVIIIHD-800 spectrometer equipped with a 3-mm TCI cryoprobe. All data were processed in NMRPipe^63^ and analyzed in NMRFAM-SPARKY^64^.

Uniformly double-labeled ^15^N/^13^C design proteins were prepared in NMR buffer as described above at final concentrations of 200 μM to 1,400 μM. Backbone HN, N, Cα, C13 and CO resonances were assigned using sequential assignment strategies^65^ via standard triple-resonance experiments with non-uniform sampling with 20% Poisson gap sampling schedule and were reconstructed with istHMS10 (http://gwagner.med.harvard.edu/intranet/hmsIST/). The following experiments were recorded: 3D HNCA (Bruker pulse sequence hncagpwg3d), 3D HNCO (Bruker pulse sequence hncogpwg3d), 3D HNCACB (Bruker pulse sequence hncacbgpwg3d) and 3D CBCACONH (Bruker pulse sequence cbcaconhgpwg3d). Acquisition times were 92 ms in ^1^H, 15 ms in ^15^N, 20 ms in ^13^CO and 10/5 ms in ^13^Cα/C13. Recycle delay was set to 1 s in all experiments, which were recorded at a ^1^H field of 800 MHz at 37 °C. To obtain through-space restraints for structure calculation, 3D amide–amide NOESY experiments (3D SOFAST _HNHAro-NHN_) were collected with 8–16 scans, 0.6-s recycle delay and 350-ms mixing time^66^. Nuclear Overhauser effect (NOE) cross-peaks were assigned manually in NMRFAM-SPARKY. NMR peak assignments were used by TALOS-N^44^ to determine secondary structure information, random coil index order parameter predictions and dihedral angle restraints toward structure calculation. Structure calculations were set up with automated Python scripts using CS-Rosetta^67,68^. We first used TALOS-N to determine psi and phi dihedral angles, and we used protein design sequences and assigned chemical shift values to pick fragments of amino acid lengths 3 and 9. We then used the protein sequence, 3mer/9mer fragments, backbone chemical shifts and amide–amide NOEs as input for the abrelax CS-Rosetta protocol (Rosetta version 3.8 and CS-Rosetta Toolbox version 3.3). From the 30,000 decoys calculated, the 10 lowest energy models were selected to represent the final NMR ensemble structure. The structure calculation was considered converged because the lowest energy models clustered within less than 2 Å from the model with the lowest energy. Final structures were validated with MolProbity.

^1^H_N_ and ^15^N ACS values were determined from peaks in 2D ^1^H-^15^N HSQC spectra using the following equation^43,69^:

$${{\rm{ACS}}}_{{\rm{i}}}=\frac{1}{N}\sum _{K=1,M}{\omega }_{k}$$

where i = ^1^H_N_ and ^15^N atoms; N is the total number of peaks picked in the HSQC spectrum; M is the total number of residues in the protein sequence; and wk is the chemical shift of the k-th resonance.

Reference ^1^H_N_ and ^15^N ACS values for primarily α-helical proteins and primary 13-sheet proteins were taken from previous reports by Mielke et al.^43,69^.

### Furin cleavage

To cleave designed proteins, 5 U of furin protease (New England Biolabs, P8077S) was combined with 30 µM design in enzyme buffer (20 mM HEPES, 1 mM CaCl_2_, 0.2 mM 13-mercaptoethanol) and incubated at 25 °C for 16 h. Cleavage reaction was used for SDS-PAGE (Any kDTM Mini-PROTEAN TGXTM Precast Protein Gels) with protein standards (Precision Plus Protein Dual Color Standards).

### Blood cell lysis assay

Hemolysis assay was performed as described previously.^70^ Single-donor washed human RBCs (Innovative Research, IWB3ALS40ML) were washed three times by spinning blood at 500*g* for 5 min and discarding supernatant until supernatant appears clear. PBS was used to resuspend the RBCs at 10% hematocrit (v/v). Blood cell lysis was carried out in a 96-well plate at a final hematocrit of 2.5%. Negative controls include PBS, cleavage buffer and cleavage buffer with 0.5 U of furin. Positive controls include 2% Triton-X-100 (Sigma-Aldrich, 9036-19-5) and 15 µM melittin (GenScript, RP20415). Designed proteins were diluted to 15 µM with PBS. Washed RBCs were added to each well and incubated at 37 °C for 1 h, after which the reaction plate was spun down at 500*g* for 5 min. Supernatant from the reaction plate was transferred to a 96-well clear-bottom microplate (Corning, 3598). Absorbance was measured at 450 nm on an Agilent BioTek Epoch 2 TSC microplate reader.

### Crystallography

All crystallization experiments were conducted using the sitting drop vapor diffusion method. Crystallization trials were set up in 200-nl drops using the 96-well plate format at 20 °C.

Crystallization plates were set up using a mosquito LCP from SPT Labtech and then imaged using UVEX microscopes and UVEX PS-256 from JAN Scientific. Diffraction quality crystals formed in a mixture of 0.1 M PCB buffer (pH 4) and 25% PEG 1500.

Diffraction data were collected at the National Synchrotron Light Source II. X-ray intensities and data reduction were evaluated and integrated using XDS^71^ and merged/scaled using Pointless/Aimless in the CCP4 program suite^72^. Structure determination and refinement starting phases were obtained by molecular replacement using Phaser^73^ using the designed model for the structures. After molecular replacement, the models were improved using phenix.autobuild^74^. Structures were refined in Phenix^74^. Model building was performed using Coot^75^. The final model was evaluated using MolProbity^76^. Data collection and refinement statistics are recorded in Table 1. Data deposition, atomic coordinates and structure factors reported in this paper have been deposited in the PDB with accession code 8VD6.

### Reporting summary

Further information on research design is available in the Nature Portfolio Reporting Summary linked to this article.

## Online content

Any methods, additional references, Nature Portfolio reporting summaries, source data, extended data, supplementary information, acknowledgements, peer review information; details of author contributions and competing interests; and statements of data and code availability are available at https://doi.org/10.1038/s41587-024-02395-w.

## Supplementary information

Supplementary Information Supplementary Figs. 1–27, Supplementary Algorithms 1–4 and Supplementary Tables 1 and 2.

Reporting Summary

Source Data Fig. 4 Unprocessed SDS-PAGE gel for Fig. 4.

## Data Availability

Atomic coordinates have been deposited in the Protein Data Bank (http://www.rcsb.org/) with accession codes 8VD6 (ref. ^77^) (Crystal structure of CAP repeat), 8VL4 (ref. ^78^) (NMR structure of MS3 parent) and 8VL3 (ref. ^79^) (NMR structure of MS2 parent). The backbone chemical shift assignments of design proteins have been deposited to the Biological Magnetic Resonance Bank with accession codes 31137 (MS3 parent) and 31136 (MS2 parent).

## Code availability

The code^80^ for this project, with the exception of training scripts, is available at https://github.com/RosettaCommons/protein_generator. For greater accessibility, we thank Simon Dürr and HuggingFace who supplied a GPU grant to run the model interactively in their browser: https://huggingface.co/spaces/merle/PROTEIN_GENERATOR.
