# Publisher Correction: Multistate and functional protein design using RoseTTAFold sequence space diffusion

**DOI:** 10.1038/s41587-024-02456-0

**Published:** 2024-10-07

**Authors:** Sidney Lyayuga Lisanza, Jacob Merle Gershon, Samuel W. K. Tipps, Jeremiah Nelson Sims, Lucas Arnoldt, Samuel J. Hendel, Miriam K. Simma, Ge Liu, Muna Yase, Hongwei Wu, Claire D. Tharp, Xinting Li, Alex Kang, Evans Brackenbrough, Asim K. Bera, Stacey Gerben, Bruce J. Wittmann, Andrew C. McShan, David Baker

**Affiliations:** 1 Department of Biochemistry, University of Washington, Seattle, WA USA; 2 Institute for Protein Design, University of Washington, Seattle, WA USA; 3 Graduate Program in Biological Physics, Structure and Design, University of Washington, Seattle, WA USA; 4 Department of Molecular Engineering, University of Washington, Seattle, WA USA; 5 Molecular & Cellular Biology, Medical Scientist Training Program, University of Washington, Seattle, WA USA; 6 Faculty of Engineering Sciences, Heidelberg University, Heidelberg, Germany; 7 School of Chemistry and Biochemistry, Georgia Institute of Technology, Atlanta, GA USA; 8 Office of the Chief Scientific Officer, Microsoft, Redmond, WA USA; 9 Howard Hughes Medical Institute, University of Washington, Seattle, WA USA

**Keywords:** Proteins, Computational models, Molecular engineering

Correction to: *Nature Biotechnology* https://doi.org/10.1038/s41587-024-02395-w, published online 25 September 2024.

In the version of the article initially published, in the key to Fig. 2f the colors were swapped and have now been amended so that “W guidance” is purple and “Unconditional” is grey. In Fig. 5a, the color of “Child B” has been corrected from pink to purple, and in Fig. 5d, "β-helix" has now been amended to "β-sheet". These corrections have been made to the HTML and PDF versions of the article.

