# Supplementary material for: Multistate and functional protein design using RoseTTAFold sequence space diffusion
Source: Nat Biotechnol. 2024 Sep 25;43(8):1288–98. doi: 10.1038/s41587-024-02395-w (PMC12339374; doi:10.1038/s41587-024-02395-w)
Supplement: Supplementary file 1 [file 41587_2024_2395_MOESM1_ESM.pdf]

nature biotechnology

Supplementary information

https://doi.org/10.1038/s41587-024-02395-w

Multistate and functional protein design using RoseTTAFold sequence space diffusion

In the format provided by the authors and unedited

Table of Contents

Table of Contents 1
Supplementary Figures 3
Supplementary Figure 1. Abstracted schematic overview of information flow through ProteinGenerator 3
Supplementary Figure 2. Sampling comparison of starting RoseTTAFold structure prediction weights and ProteinGenerator weights 4
Supplementary Figure 3. Inference benchmarks 5
Supplementary Figure 4. Amino acid distributions and secondary structure propensities sampling from different GMMs 6
Supplementary Figure 5. Unconditional benchmark comparison 7
Supplementary Figure 6. Representative unfiltered unconditional samples from a variety of noise distributions and lengths 8
Supplementary Figure 7. Amino acid distribution of ProteinGenerator compared to native 9
Supplementary Figure 8. AlphaFold2 metrics for motif scaffolding problems from PDB 10
Supplementary Figure 9. Experimental validation of unconditional designs 11
Supplementary Figure 10. Amino acid distributions of proteins generated with amino acid compositional bias 12
Supplementary Figure 11. Deconvolved mass spectroscopy of design cys_1 13
Supplementary Figure 12. Deconvolved mass spectroscopy of design cys_2 14
Supplementary Figure 13. Deconvolved mass spectroscopy of design cys_3 15
Supplementary Figure 14. Deconvolved mass spectroscopy of design cys_4 16
Supplementary Figure 15. Deconvolved mass spectroscopy of design cys_5 17
Supplementary Figure 16. Secondary structure conditioning results in strong self consistency to DSSP of the parent 18
Supplementary Figure 17: Repeat Protein Examples 19
Supplementary Figure 18. Deconvolved mass spectroscopy of design D12 20
Supplementary Figure 19. Picture of red blood cell (RBC) hemolysis experiment after incubation with design D12 21
Supplementary Figure 20. AF2 models of barcoded designs 22
Supplementary Figure 21. Reconstructed barcode traces compared to pooled SEC trace 23
Supplementary Figure 22. Barcoded design AF2 models and corresponding SEC traces when individually expressed, pooled expression followed by MS analysis , pooled library 24
Supplementary Figure 23. Comparison of AF2 pLDDT among designed multistate proteins 25
Supplementary Figure 24. Computational models and experimental characterization

of multistate sequence families MS1-4 26
Supplementary Figure 25. Empirical cumulative distribution function plots for ProteinGenerator and bayesian optimization with upper confidence bound acquisition function 27
Supplementary Figure 26. Sequence logo plots of designs in top 1% of full mutational landscape of GB1 and those sampled by ProteinGenerator with iterative guidance 28
Supplementary Figure 27. Percent identity of all experimentally tested designs to Uniref90 based on design task. 29
Supplementary Algorithms 30
Algorithm 1. ProteinGenerator finetuning procedure 31
Algorithm 2. ProteinGenerator inference procedure 32
Algorithm 3. Amino acid bias procedure 33
Algorithm 4. Multistate inference procedure 34
Supplementary Tables 35
Supplementary Table 1: Inference timesteps, filtering criteria, success rates, and conditioning information for all experimentally tested design tasks 35
Supplementary Table 2: PG runtime to generate one protein on an A5000 GPU in 50 timesteps 36

Supplementary Figures

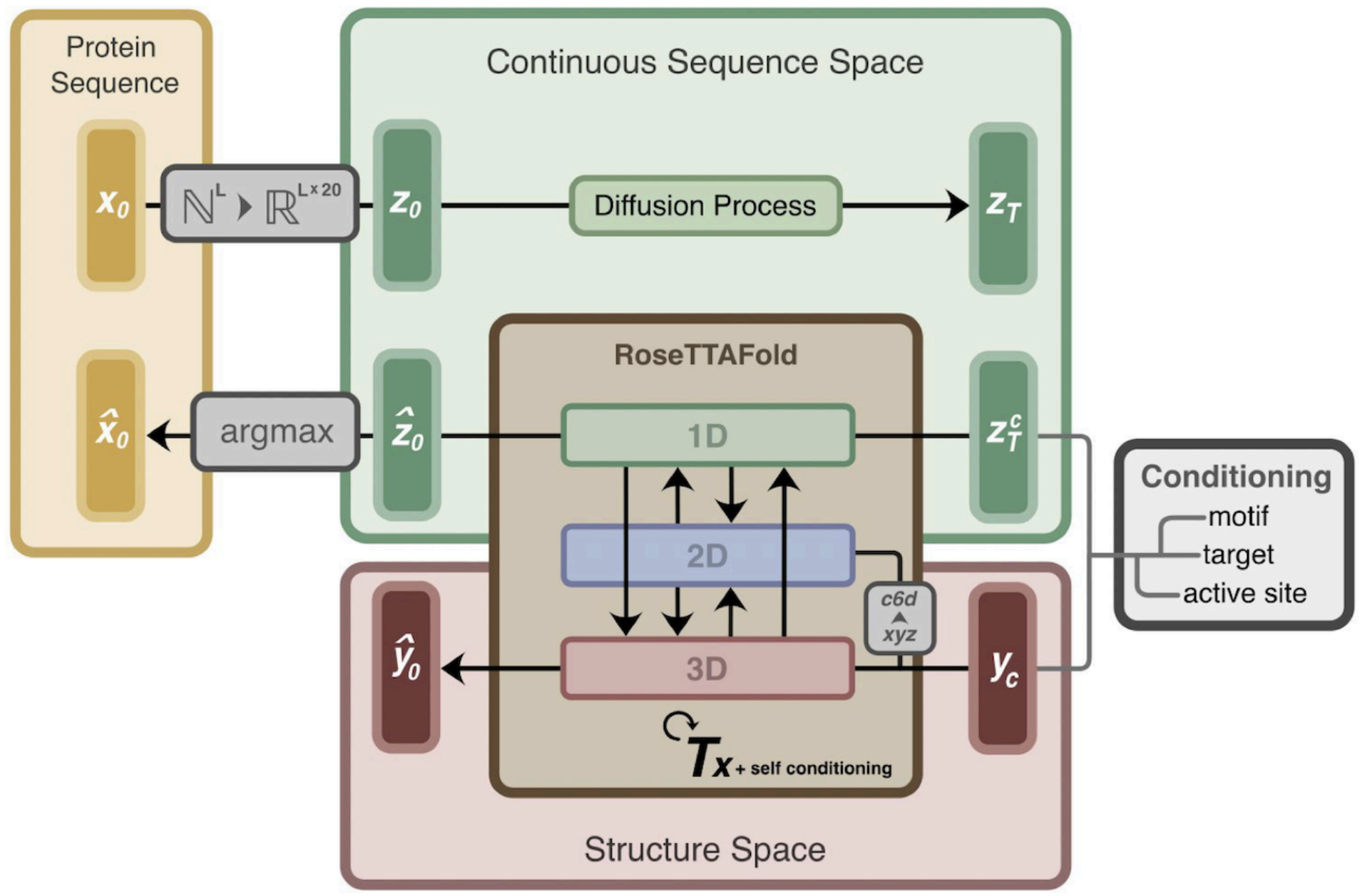

Supplementary Figure 1. Abstracted schematic overview of information flow through ProteinGenerator. Protein sequences on the left are embedded into the “continuous” space by a one-hot transformation. Diffusion noising process then takes place in the continuous space. RoseTTAFold architecture takes in the noised sequence (Xt) and structural constraints (Yc), mixes the embeddings and predicts sequence logits and a structure. Argmaxing over the predicted sequence logits maps the predicted logits back to the natural protein space.

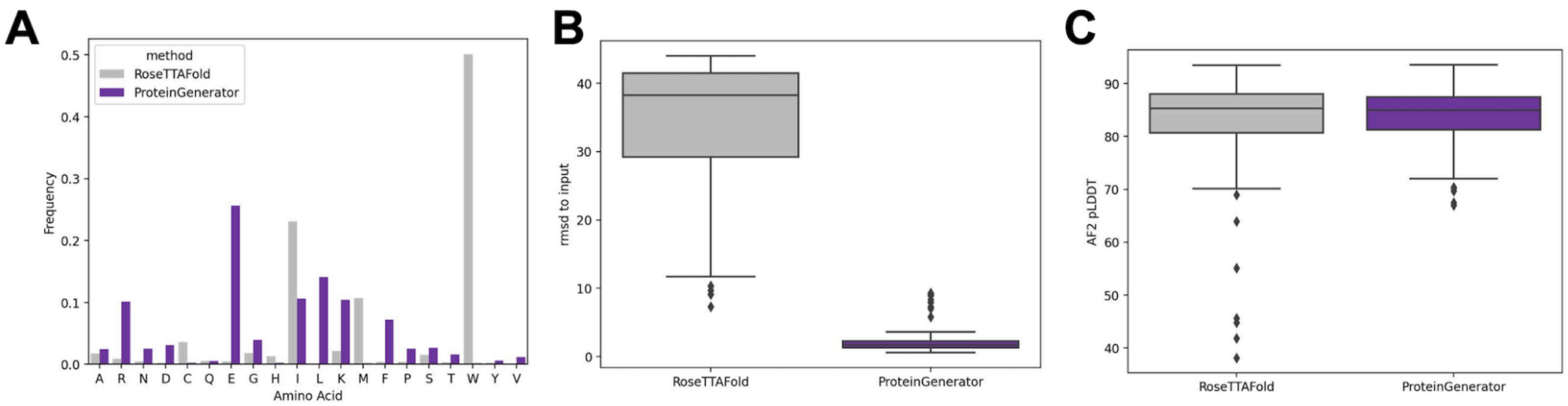

Supplementary Figure 2. Sampling comparison of starting RoseTTAFold structure prediction weights (gray) and ProteinGenerator weights (purple). All box plots boundaries indicate upper and lower quartiles, whiskers indicate the nearest quartile + 1.5*inter quartile range, center line is median (length = 125 residues, *n* = 100 designs per model). (A) Amino acid distributions in sampled designs. (B) RMSD of AF2 model to design. (C) AF2 pLDDT of designs generated.

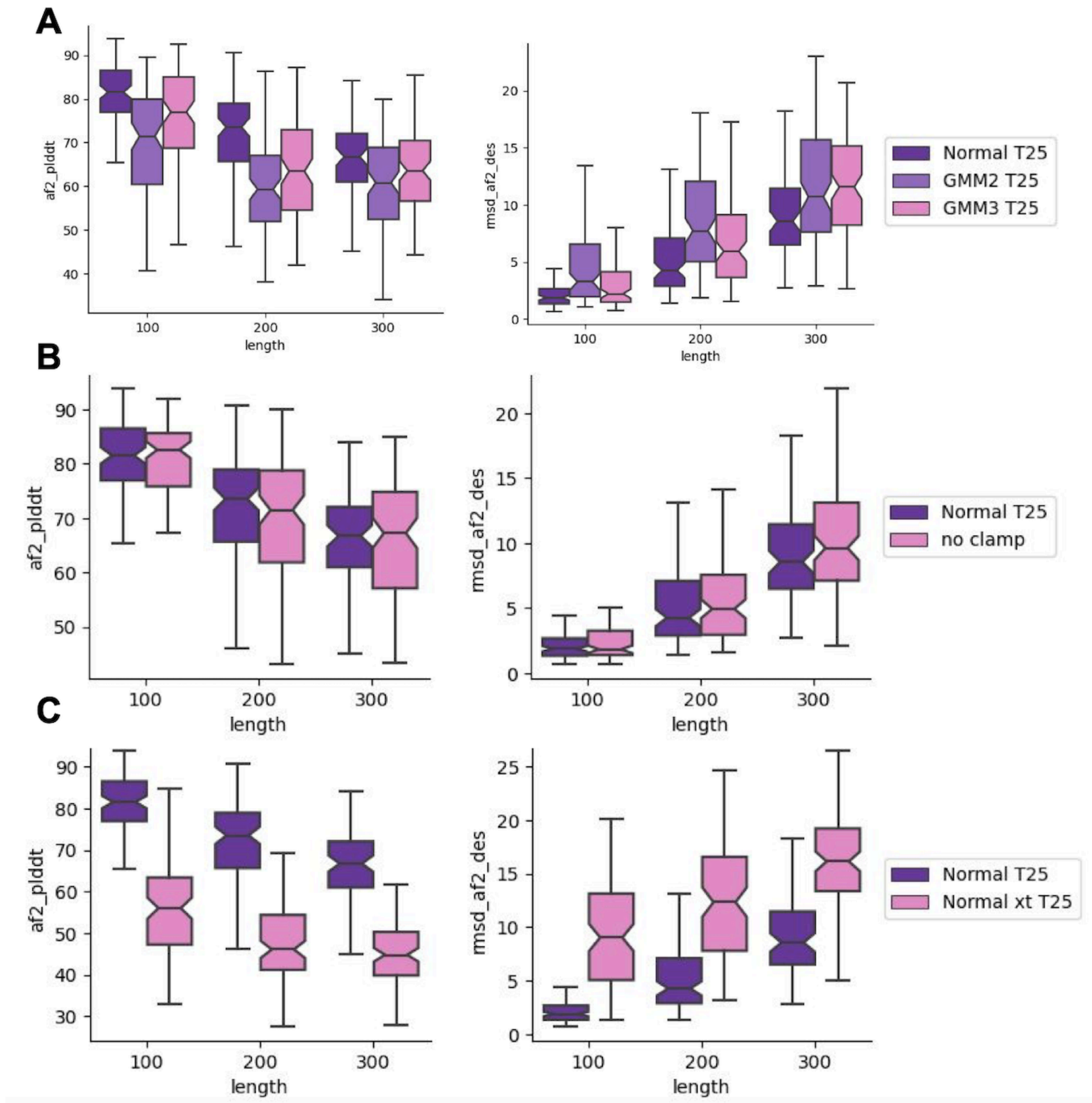

Supplementary Figure 3. Inference benchmarks. All box plots boundaries indicate upper and lower quartiles, whiskers indicate the nearest quartile + 1.5*inter quartile range, center line represents median (*n* = 100 designs per length). (A) Boxplot of AF2 pLDDT of sequences from model clustered by length on the left, right RMSD of AF2 model to design. Normal refers to sampling from a normal gaussian, GMM2 and GMM3 refer to sampling noise from multimodal distributions with gaussians centered at -1,1 and -1,0,1 respectively. (B) Boxplot of AF2 pLDDT with clamp (-3,3) applied post sampling x_{t-1} and no clamp, right side is RMSD of AF2 model to design. (C) Comparison of sampling with and without conditioning on x_t when sampling x_{t-1}, AF2 pLDDT right, AF2 RMSD to design left.

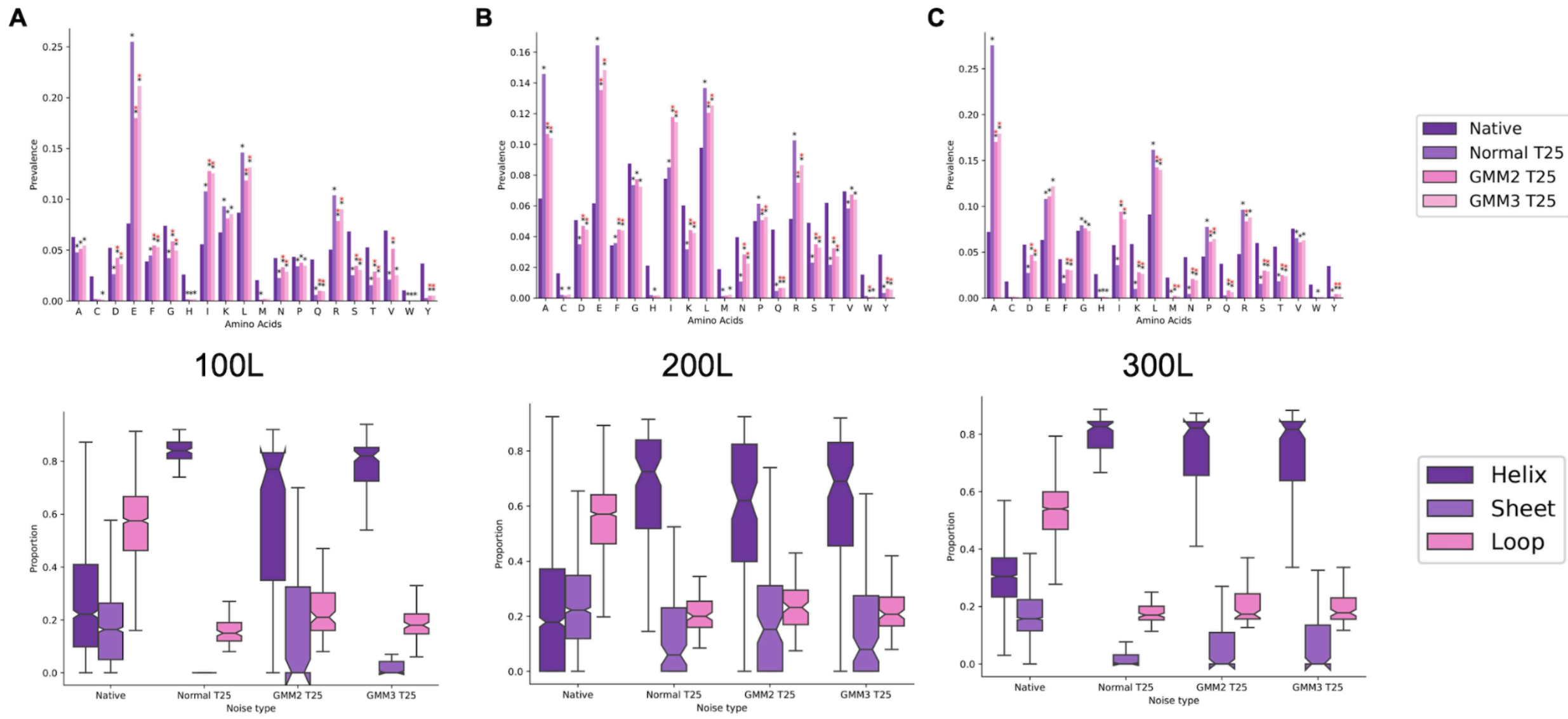

Supplementary Figure 4. Amino acid distributions and secondary structure propensities for (A) 100AA, (B) 200AA, and (C) 300AA length proteins when sampling from normally distributed noise, GMM2, or GMM3. GMM2 and GMM3 refer to sampling noise from multimodal distributions with gaussians centered at -1,1 and -1,0,1 respectively. Black and red asterisk indicate significant difference between amino acid prevalences of proteins generated by PG and native proteins or between amino acid prevalences of proteins generated by PG sampling from GMM2 or GMM3 and proteins generated by PG sampling from normally distributed noise (Welch's t-test, $\alpha = 0.05$). All box plots boundaries indicate upper and lower quartiles, whiskers indicate the nearest quartile + 1.5*inter quartile range centerline is median (n = 100 designs per length). PG likely generates more alpha-helical designs compared to native likely due to the low contact order of alpha-helices and ease of folding. Forming beta-sheet secondary structure requires the formation of more long range contacts the model is able to form, but without any conditioning information alpha-helical formation is more likely.

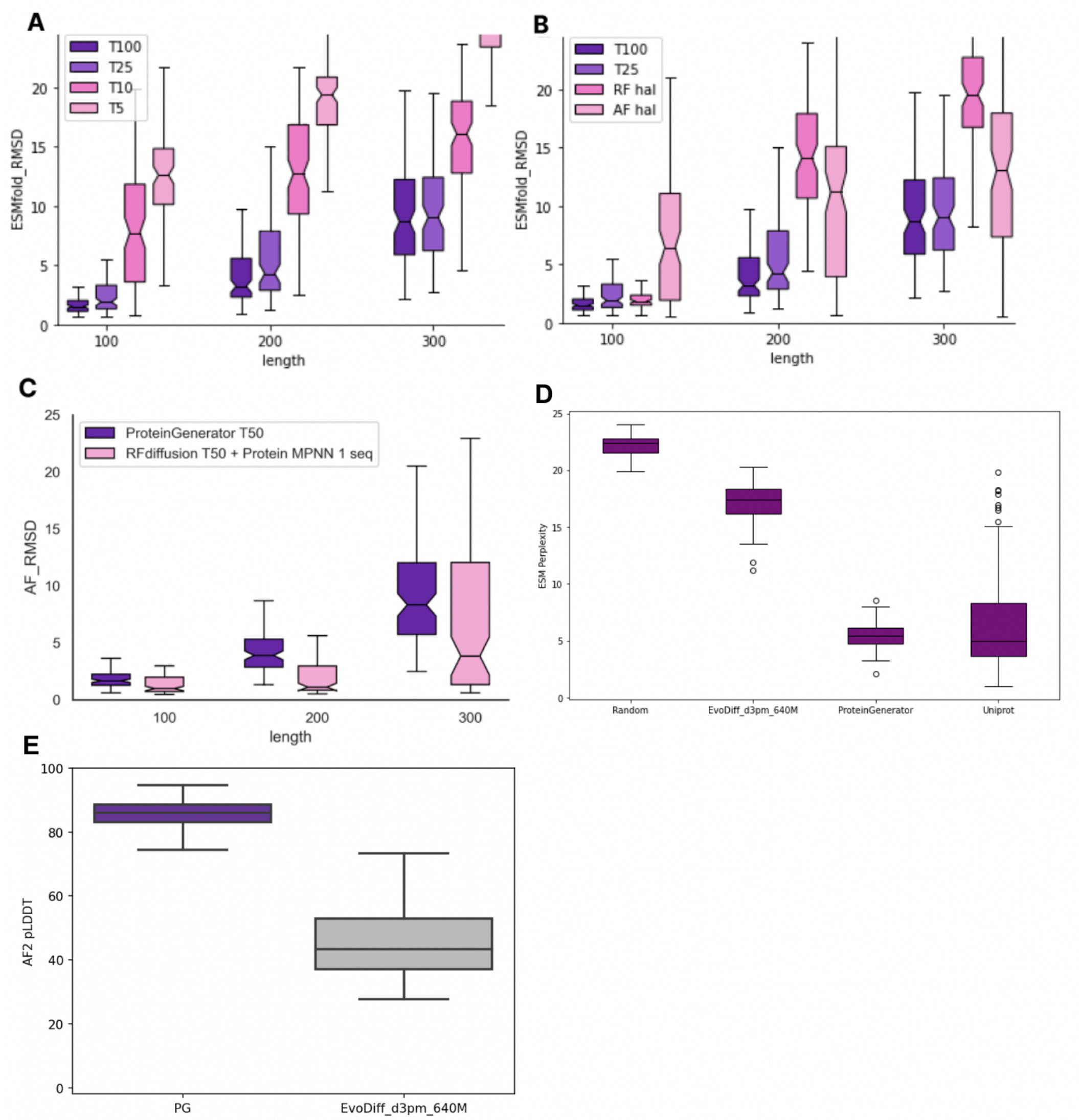

Supplementary Figure 5. Unconditional benchmark comparison. All box plots boundaries indicate upper and lower quartiles, whiskers indicate the nearest quartile + 1.5*inter quartile range, center line is median. (A) ProteinGenerator ESMFold RMSD to design for $n = 100$ unconditional proteins clustered by length and number of steps (T = [5,10,25,100]). (B) ESMFold RMSD to design comparison of ProteinGenerator co-sequence-structure generation to RoseTTAFold and AlphaFold2 hallucination both methods, with the exception of RF hal at length 100 (p=0.06), are significantly worse than PG per length ($p << 0.05$, two-sided Mann-Whitney U test, n = 100 designs per length). (C) AF2 model RMSD to design comparison between RoseTTAFold based diffusion model approaches, ProteinGenerator in purple and RF diffusion with 1 proteinMPNN designed sequence in pink (($p << 0.05$, two-sided Mann-Whitney U test, n = 100 designs per length)). (D) ESM perplexity of proteins generated randomly, with EvoDiff[25], PG, and sampled from Uniprot[24] (Length = 100, n = 75 designs each). PG

designs proteins with significantly lower ESM perplexity than EvoDiff and randomly generated sequences (two-sided Mann-Whitney U test, $p << 0.05$ across both groups compared to PG), while the difference between PG and Uniprot sequences is insignificant (two-sided Mann-Whitney U test, $p > 0.05$, $p=0.36$). (E) Comparison of AF2 pLDDT for 100 amino acid length unconditional generations from PG and EvoDiff (two-sided Mann-Whitney U test, $p << 0.05$).

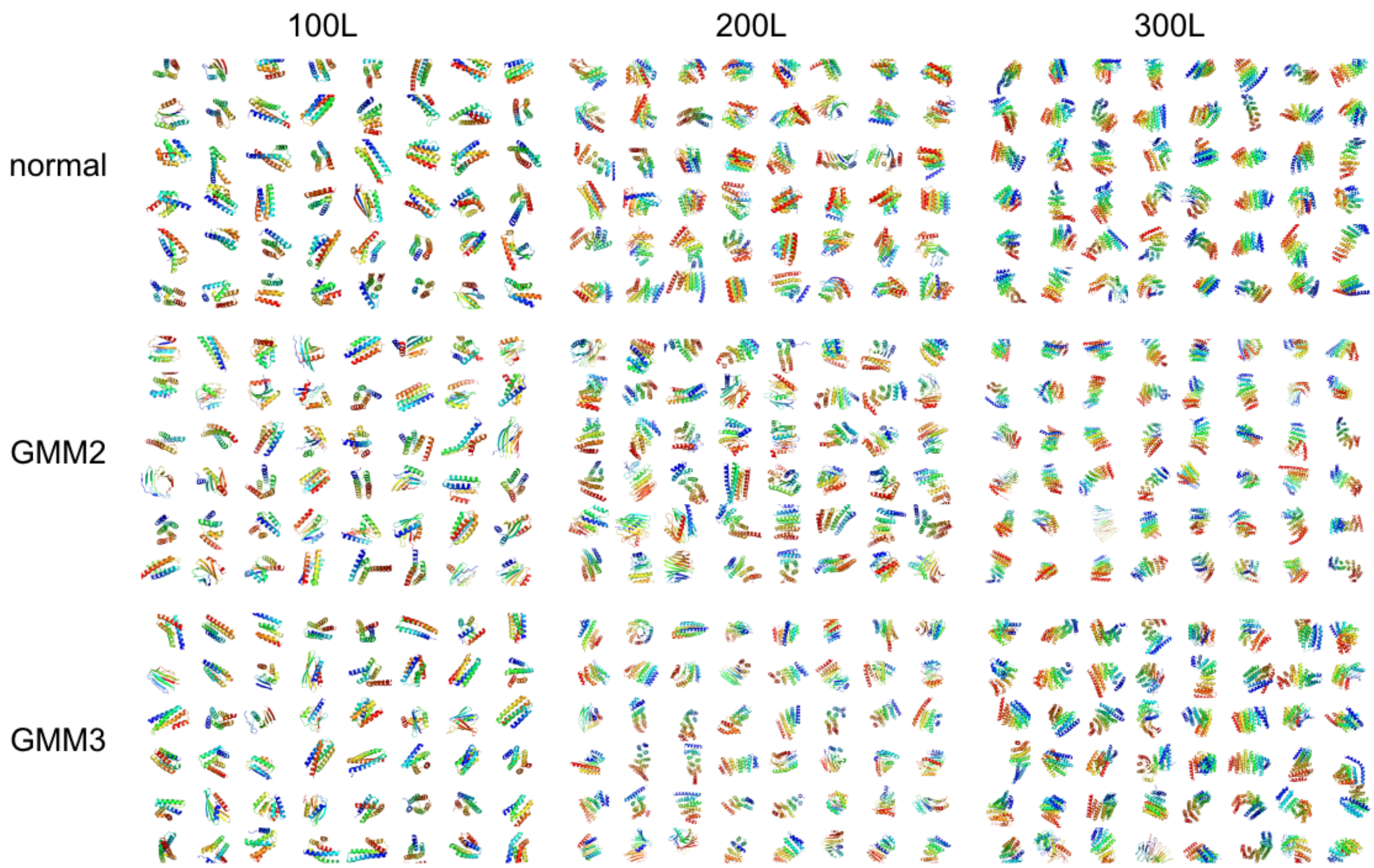

Supplementary Figure 6. Representative unfiltered unconditional samples from a variety of noise distributions and lengths (100, 200, 300). Designs are colored in a rainbow spectrum from N to C termini.

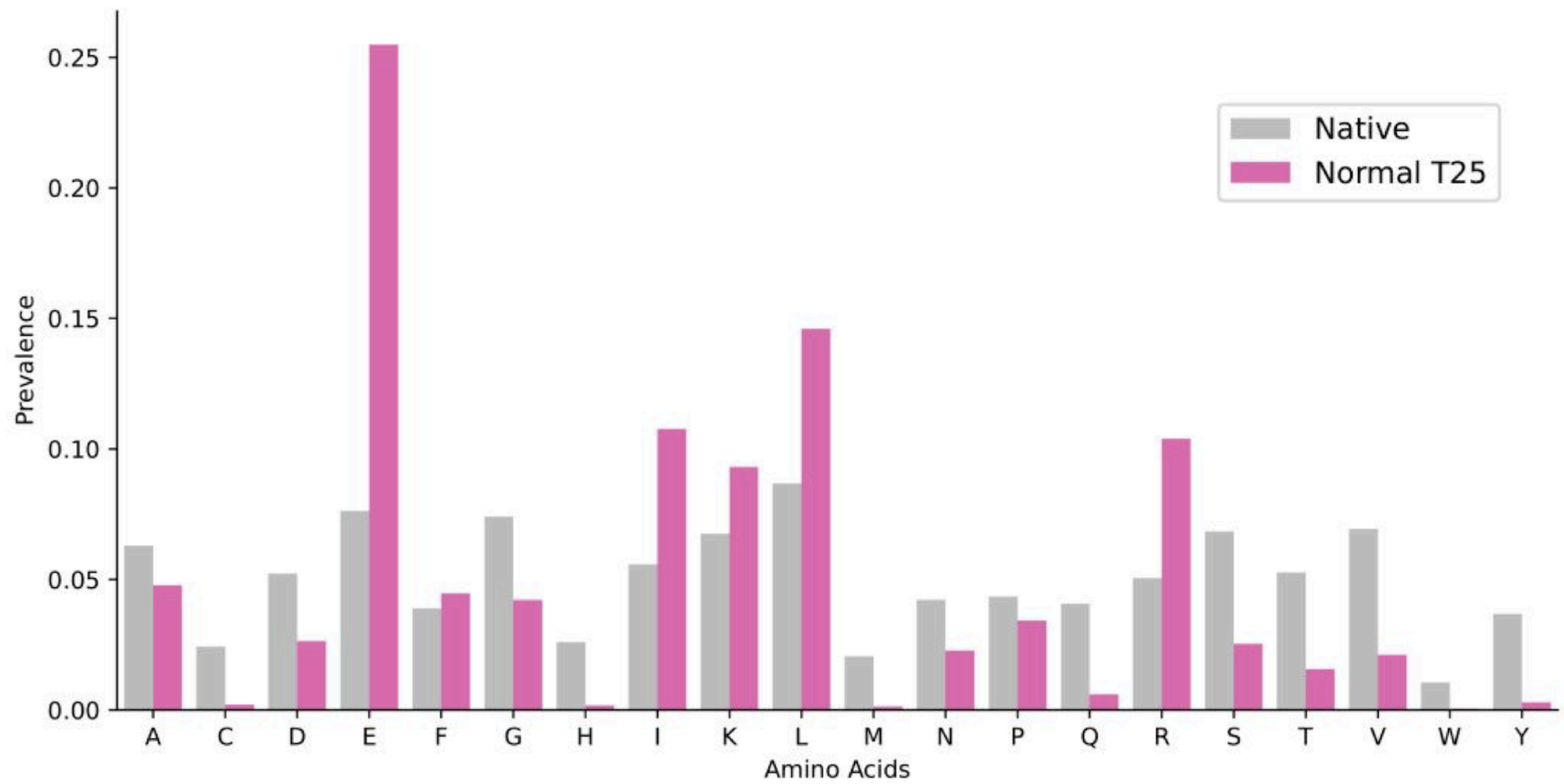

Supplementary Figure 7. Amino acid distribution of ProteinGenerator compared to native (length = 100 residues per design, *n* = 100 designs) .

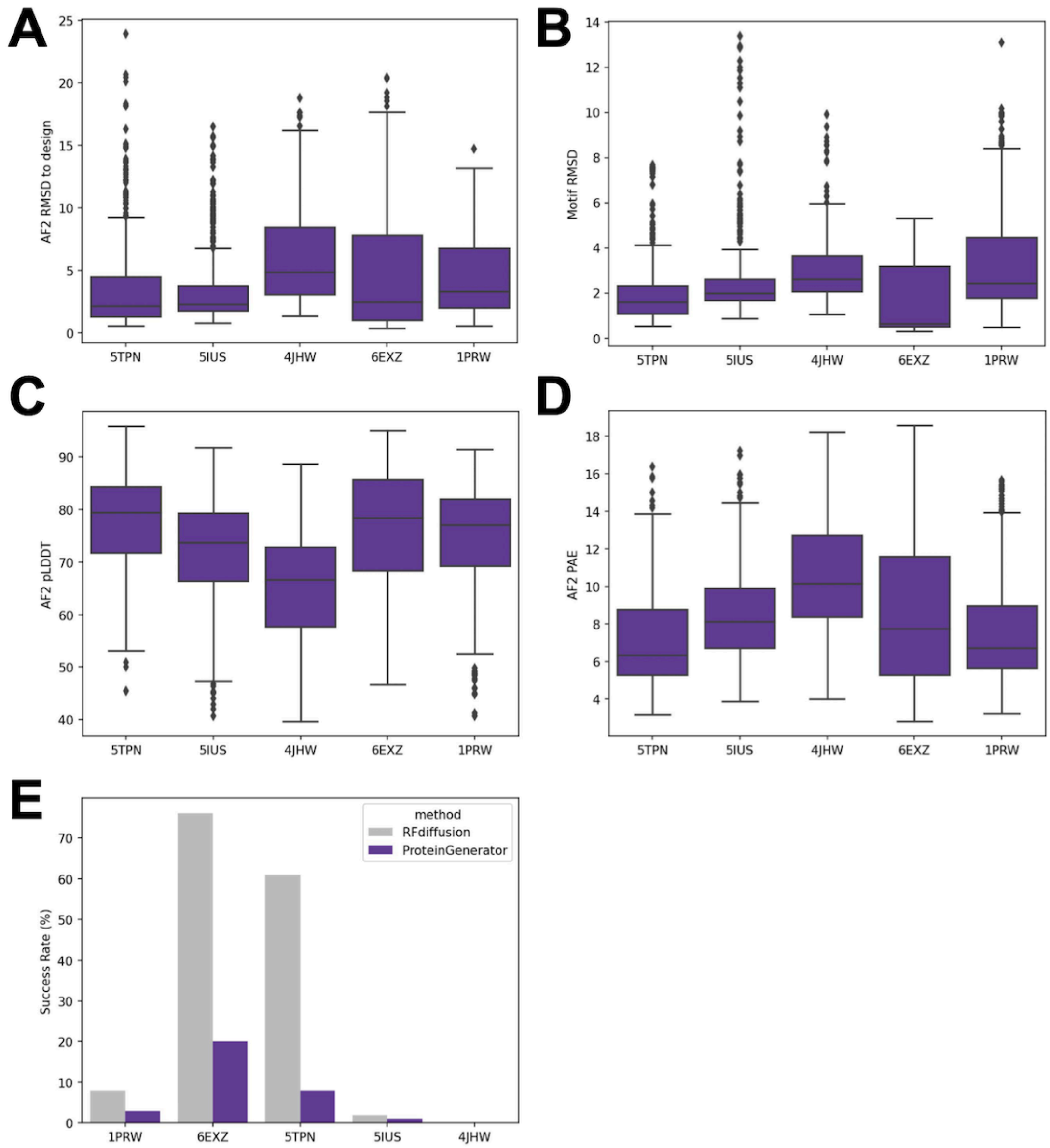

Supplementary Figure 8. AlphaFold2 metrics for motif scaffolding problems from PDB. All box plots boundaries indicate upper and lower quartiles, whiskers indicate the nearest quartile + 1.5*inter quartile range, center line is median (*n* = 200 per motif problem). (A) AF2 model RMSD to design, (B) AF2 model RMSD to original motif structure, (C) AF2 pLDDT of designs, (D) AF2 predicted aligned error (pAE) for designs. (E) Comparison with RFdiffusion + 8 MPNN sequences success rate (RMSD to design < 2A, motif RMSD < 1A, pAE < 5). The following contig arguments were used to run motif scaffolding benchmark: **1PRW** - contigs 8-20,A21-31,16-25,A56-67,8-20, **6EXZ** -contigs 0-95,A28-42,0-95, **5TPN** - contigs 10-40,A163-181,10-40, **5IUS** - contigs 0-30,A119-140,15-40,A63-82,0-30, **4JHW** - contigs 0-25,F196-212,15-30,F63-69,10-25.

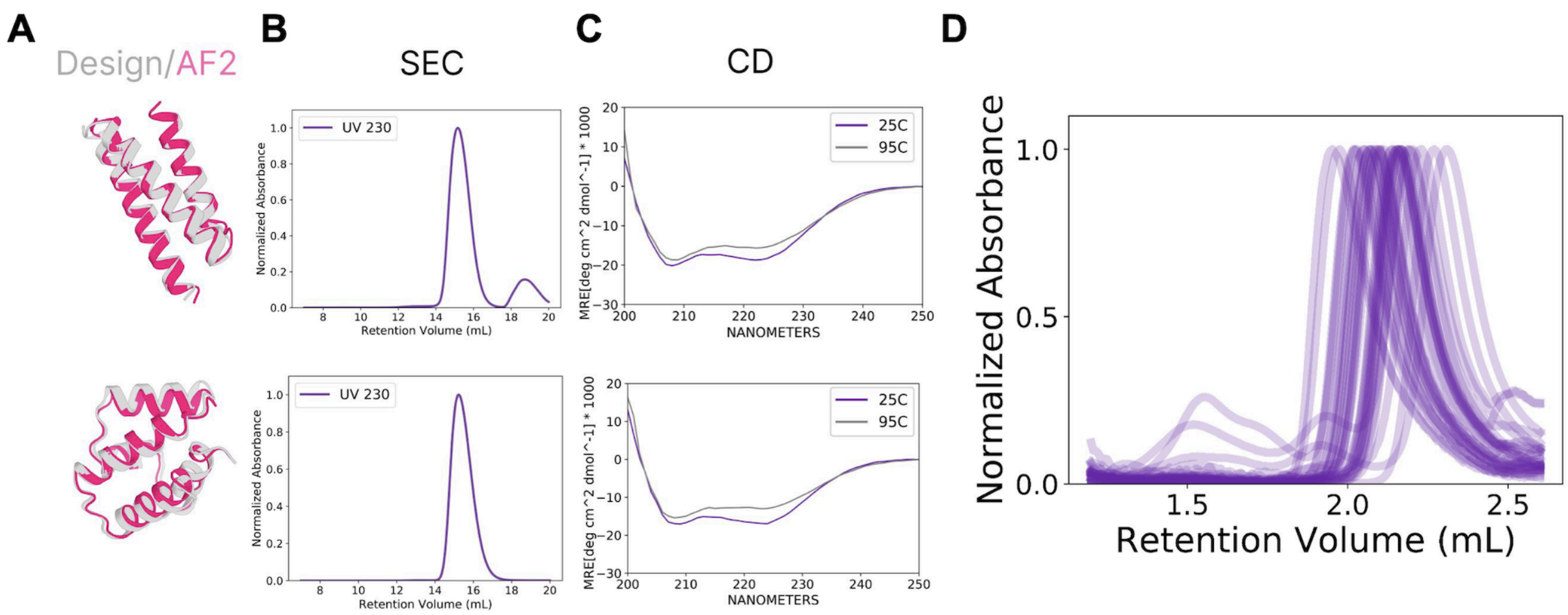

Supplementary Figure 9. Experimental validation of unconditional designs. (A) AlphaFold2 models in pink overlaid with design model in gray. (B) SEC elution profiles of designed proteins appear monomeric. (C) CD spectra of designs appear helical. (D) Overlay of expressed unconditional design SEC traces on Superdex S75 5/150 GL.

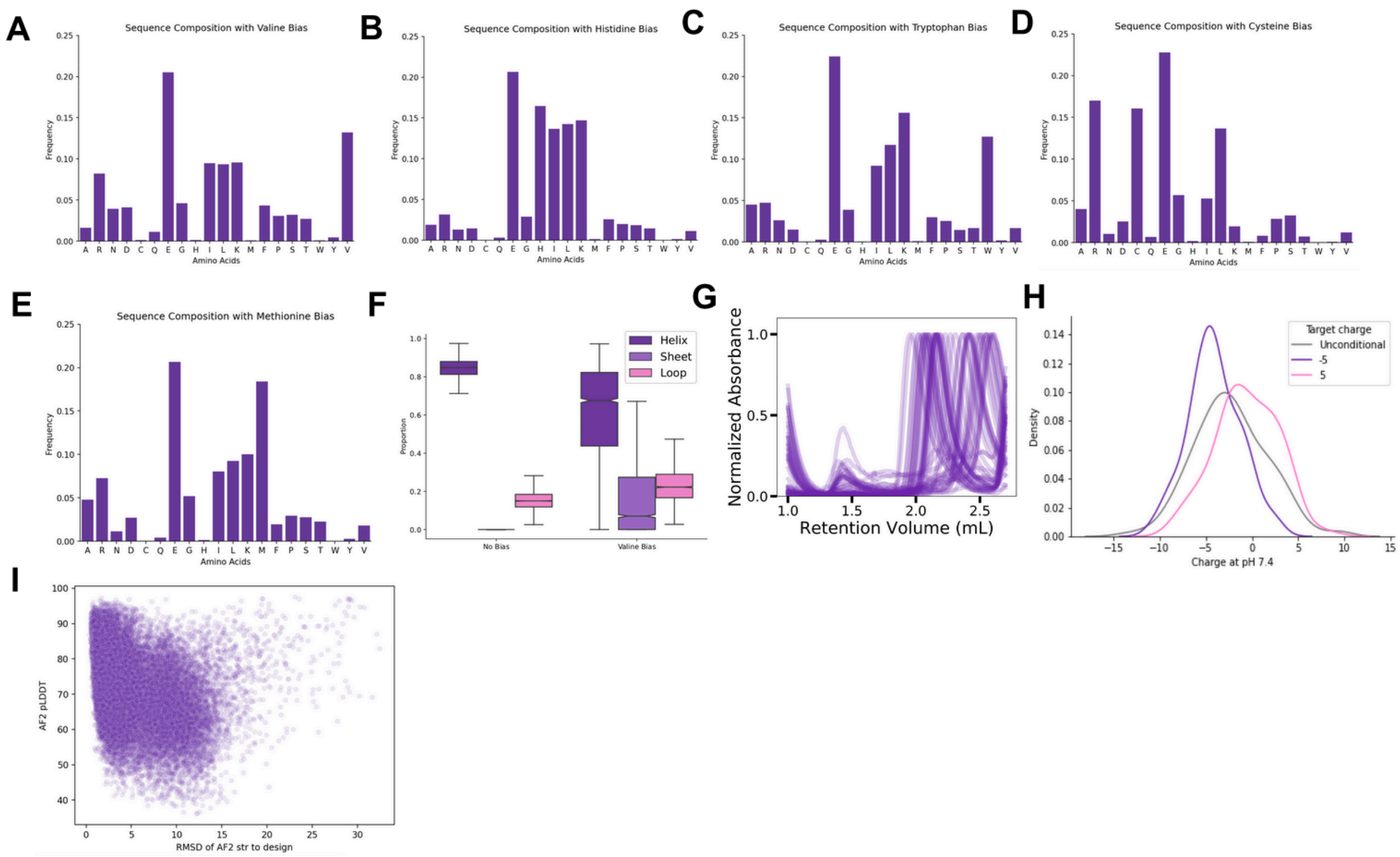

Supplementary Figure 10. Biasing for specific amino acids results in increased frequency of the specified residue in generated proteins. Amino acid distributions of proteins generated with amino acid compositional bias for (A) valine, (B) histidine, (C) tryptophan, (D) cysteine, and (E) methionine. (*n* = 200 designs per amino acid bias) (F) Secondary structure profile of unconditional generation on the left and valine biased composition on the right. Designs with valine bias tend to have more beta-sheet formation as seen in nature. Box plots boundaries indicate upper and lower quartiles, whiskers indicate the nearest quartile + 1.5*inter quartile range, center line is median (*n* = 200 designs per amino acid bias). (G) Overlay of expressed amino acid biased SEC traces Superdex S75 5/150 GL. (H) *In silico* distribution of proteins generated with net charge potential. (I) Scatterplot of AF2 pLDDT and RMSD to design for amino acid bias proteins.

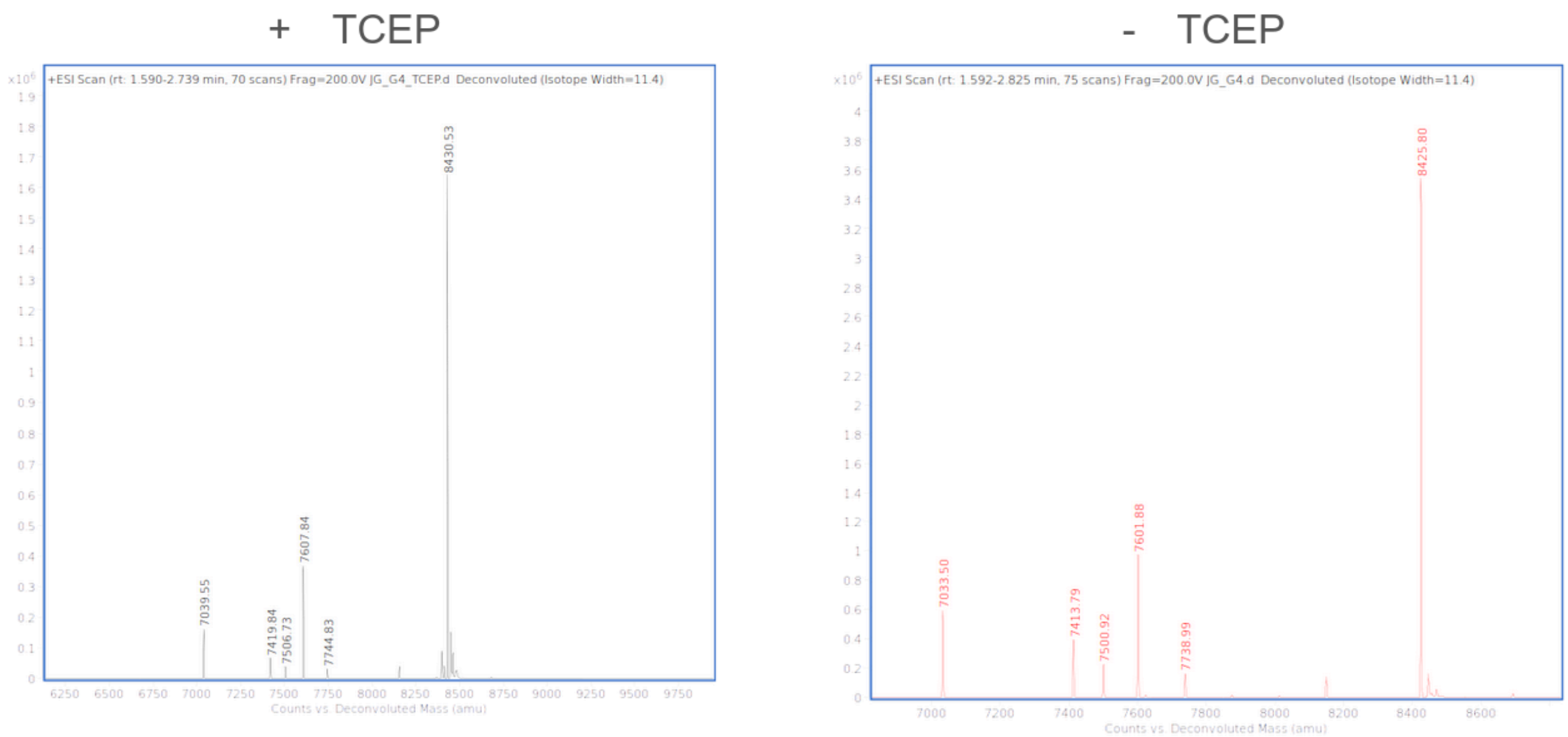

Supplementary Figure 11. Deconvolved mass spectroscopy of design cys_1 incubated with (left) or without (right) the reducing agent TCEP.

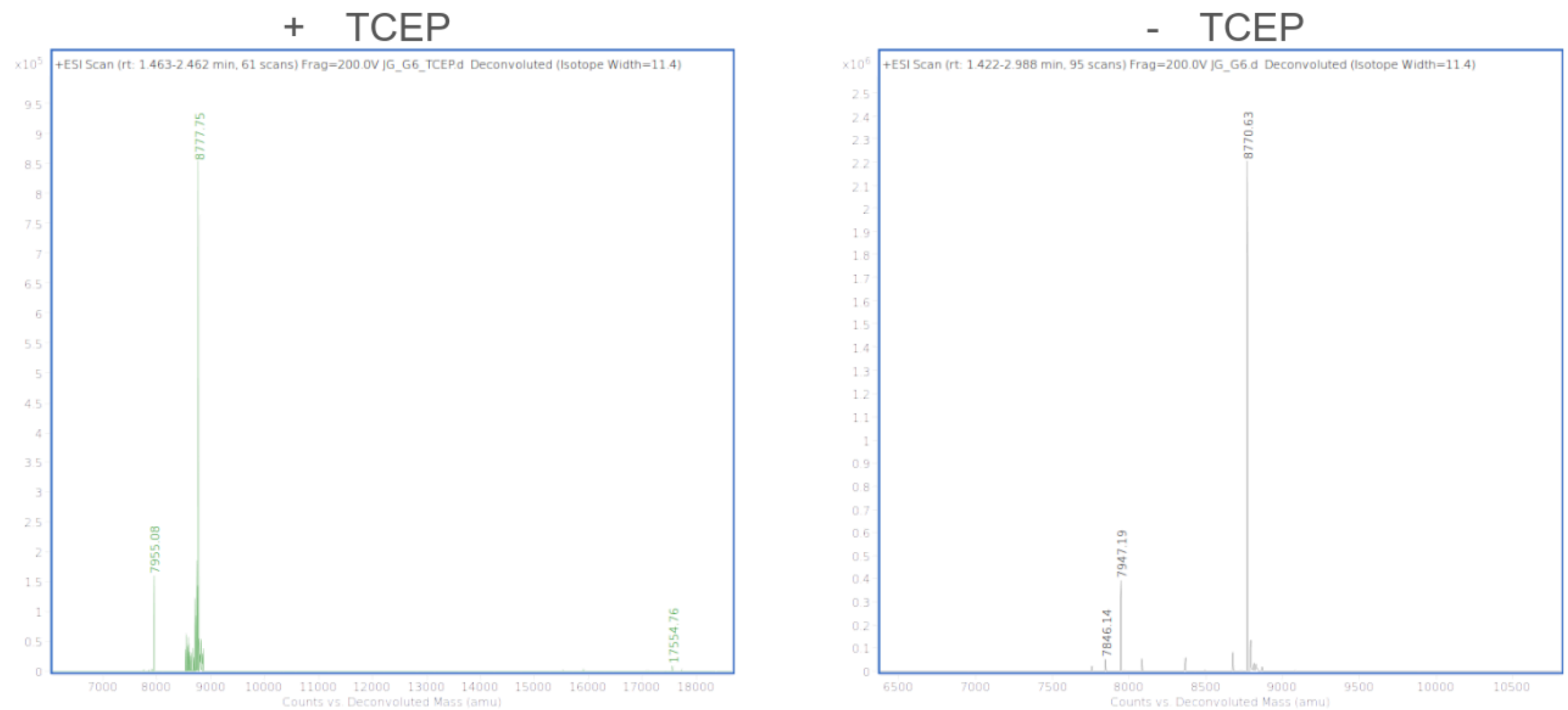

Supplementary Figure 12. Deconvolved mass spectroscopy of design cys_2 incubated with (left) or without (right) the reducing agent TCEP.

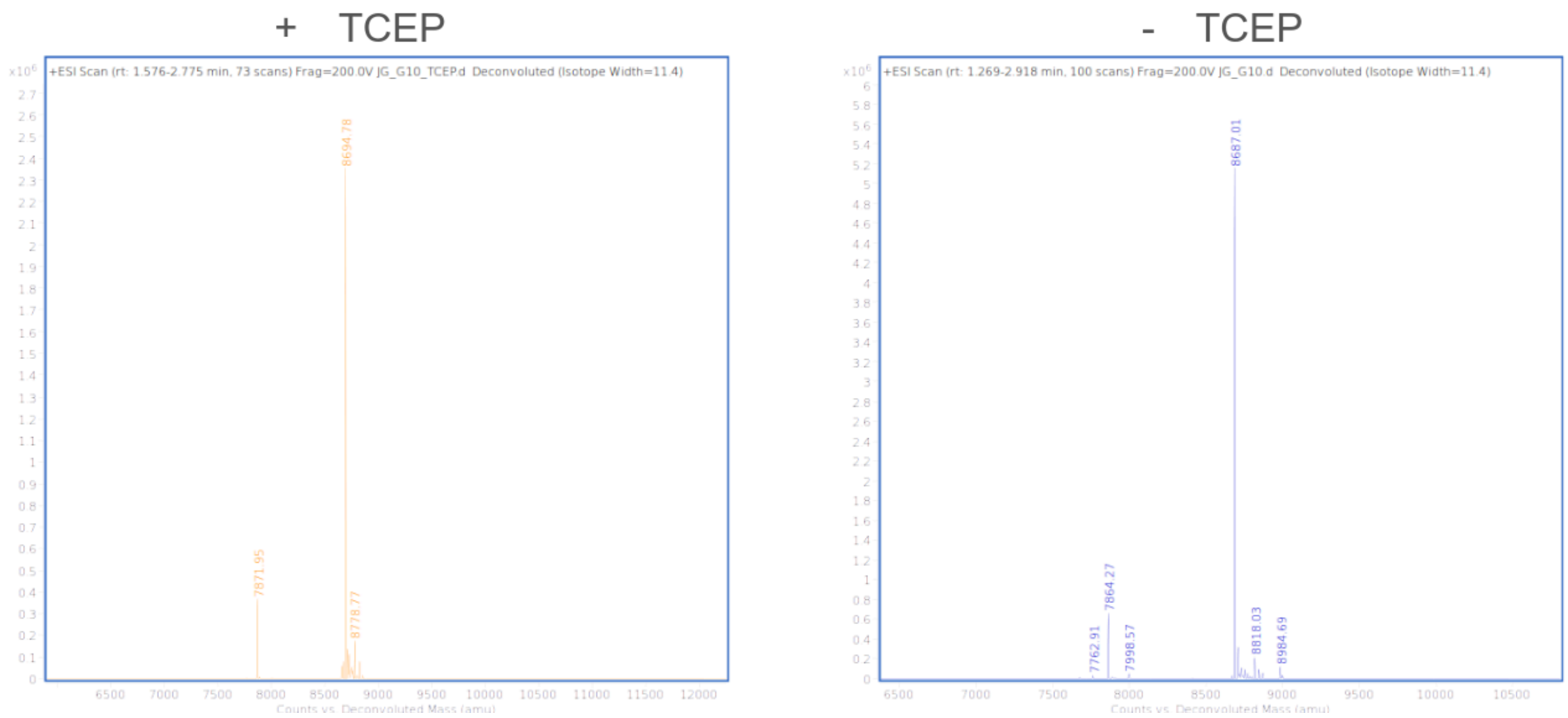

Supplementary Figure 13. Deconvolved mass spectroscopy of design cys_3 incubated with (left) or without (right) the reducing agent TCEP.

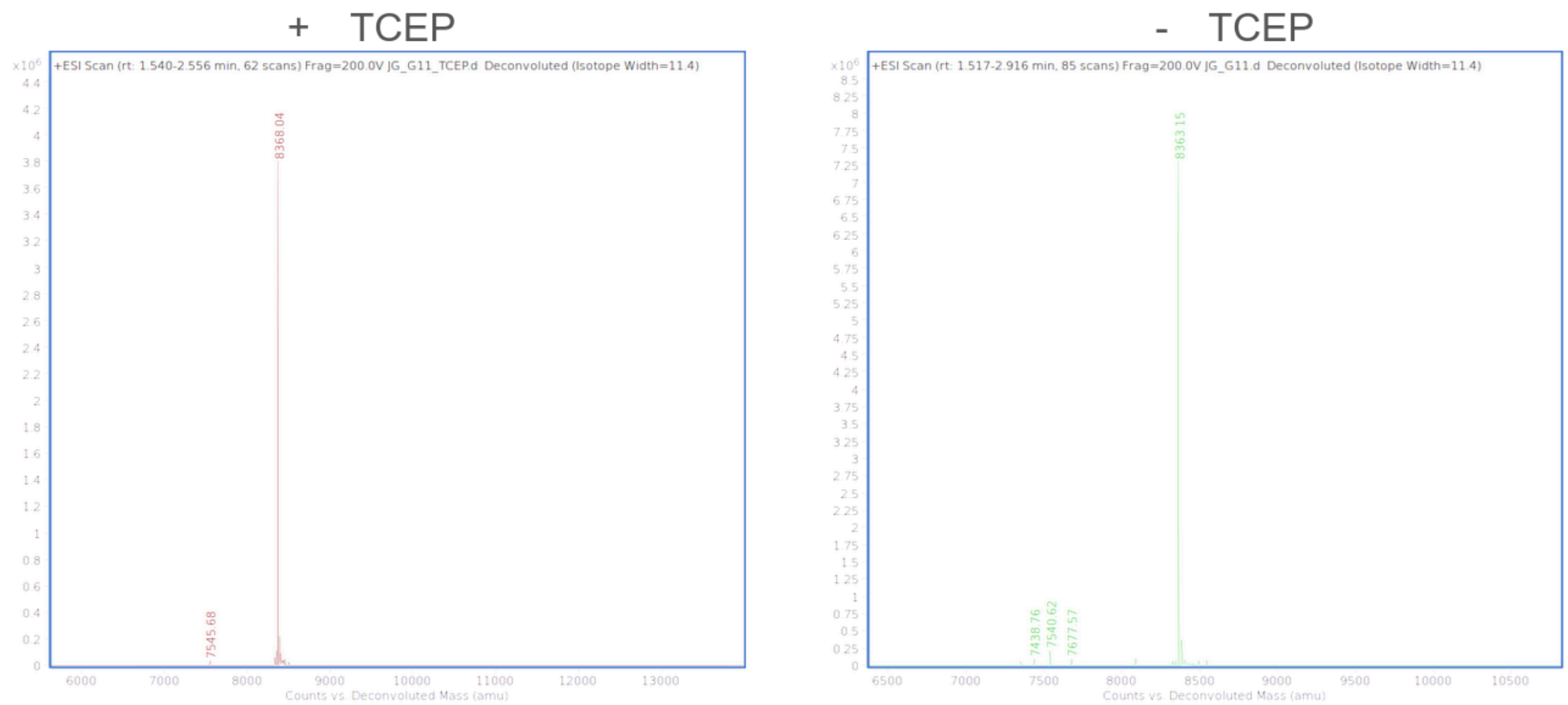

Supplementary Figure 14. Deconvolved mass spectroscopy of design cys_4 incubated with (left) or without (right) the reducing agent TCEP.

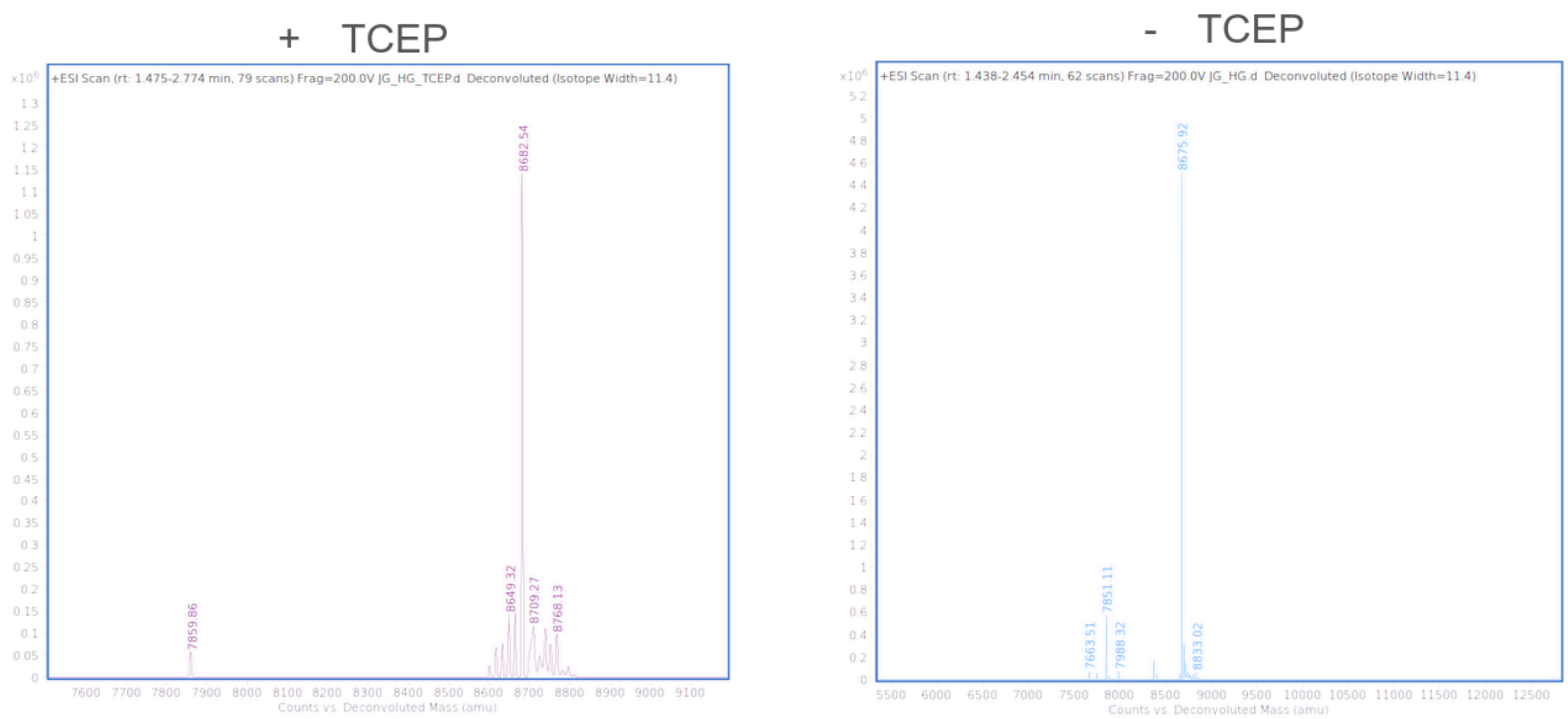

Supplementary Figure 15. Deconvolved mass spectroscopy of design cys_5 incubated with (left) or without (right) the reducing agent TCEP.

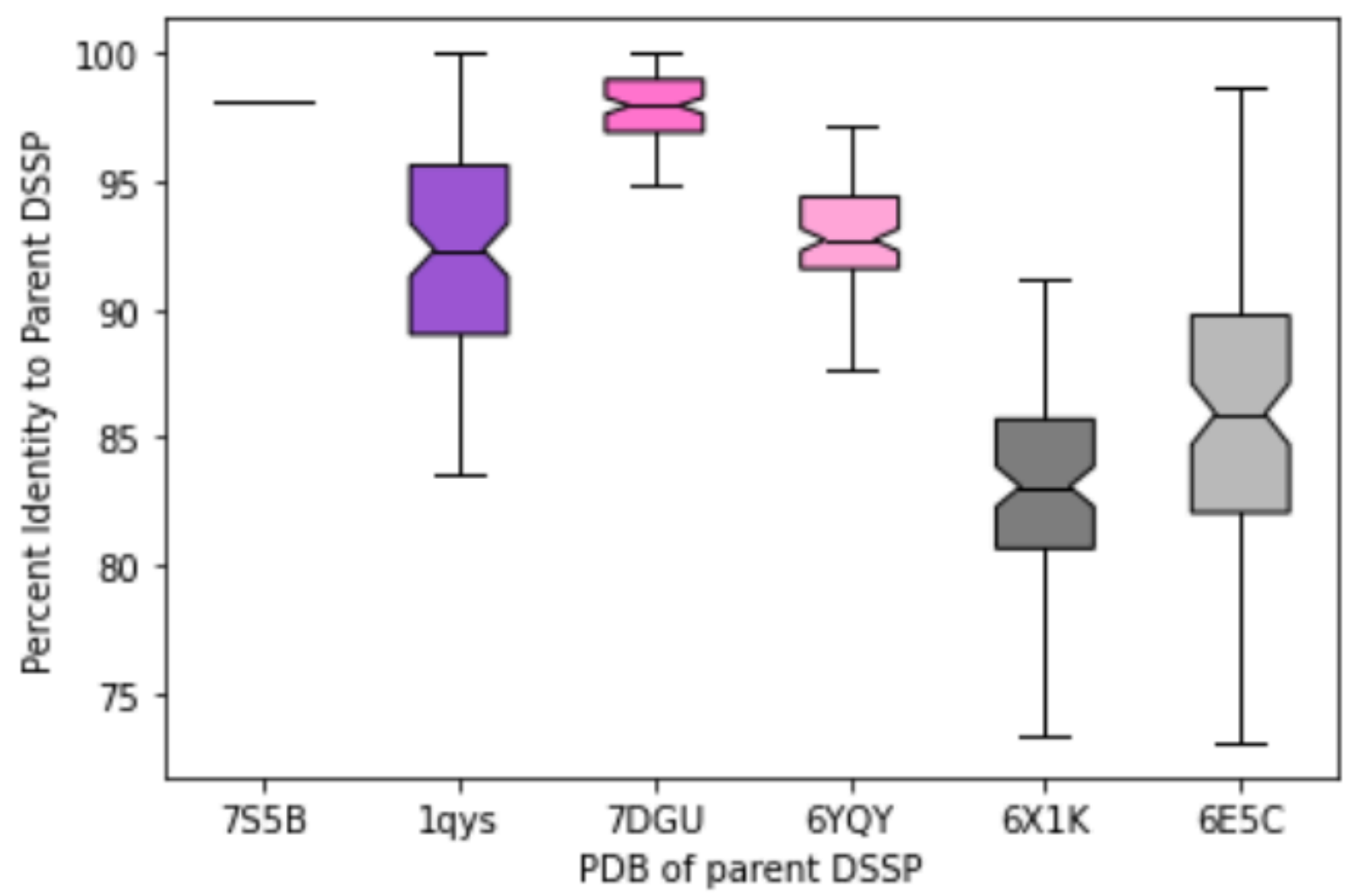

Supplementary Figure 16. Secondary structure conditioning results in strong self consistency to DSSP of the parent. Boxplot of percent identity of design secondary structure compared to parent. Box plots boundaries indicate upper and lower quartiles, whiskers indicate the nearest quartile + 1.5*inter quartile range, center line is median (*n* = 100 designs per parent pdb).

A

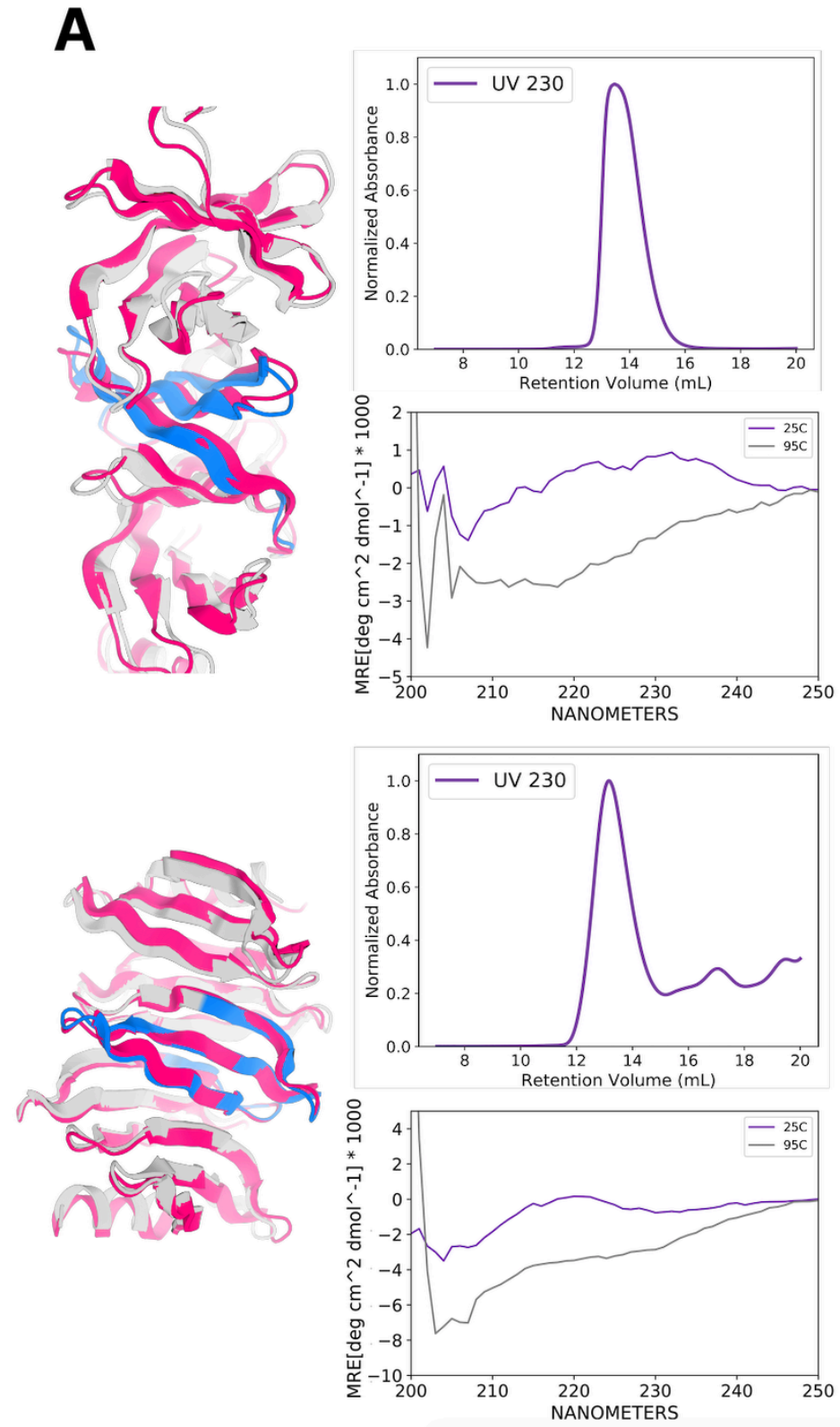

B

	CD RC_E8	CD RC_F11
**Helix**	1.0	2.7
regular	0.2	1.2
distorted	0.8	1.5
**Antiparallel**	40.7	26.8
left-twisted	0.0	0.0
relaxed	24.3	12.2
right-twisted	16.4	14.6
**Parallel**	0.0	0.0
**Turn**	14.0	17.9
**Others**	44.3	52.7

Supplementary Figure 17: Repeat Protein Examples (A) Repeat design (gray) structures overlaid with AF2 predictions (pink) with asymmetric unit (blue) displayed. SEC indicates primarily monomeric species, while CD indicates primarily beta secondary structure. RC_E8 top, RC_F11 bottom.

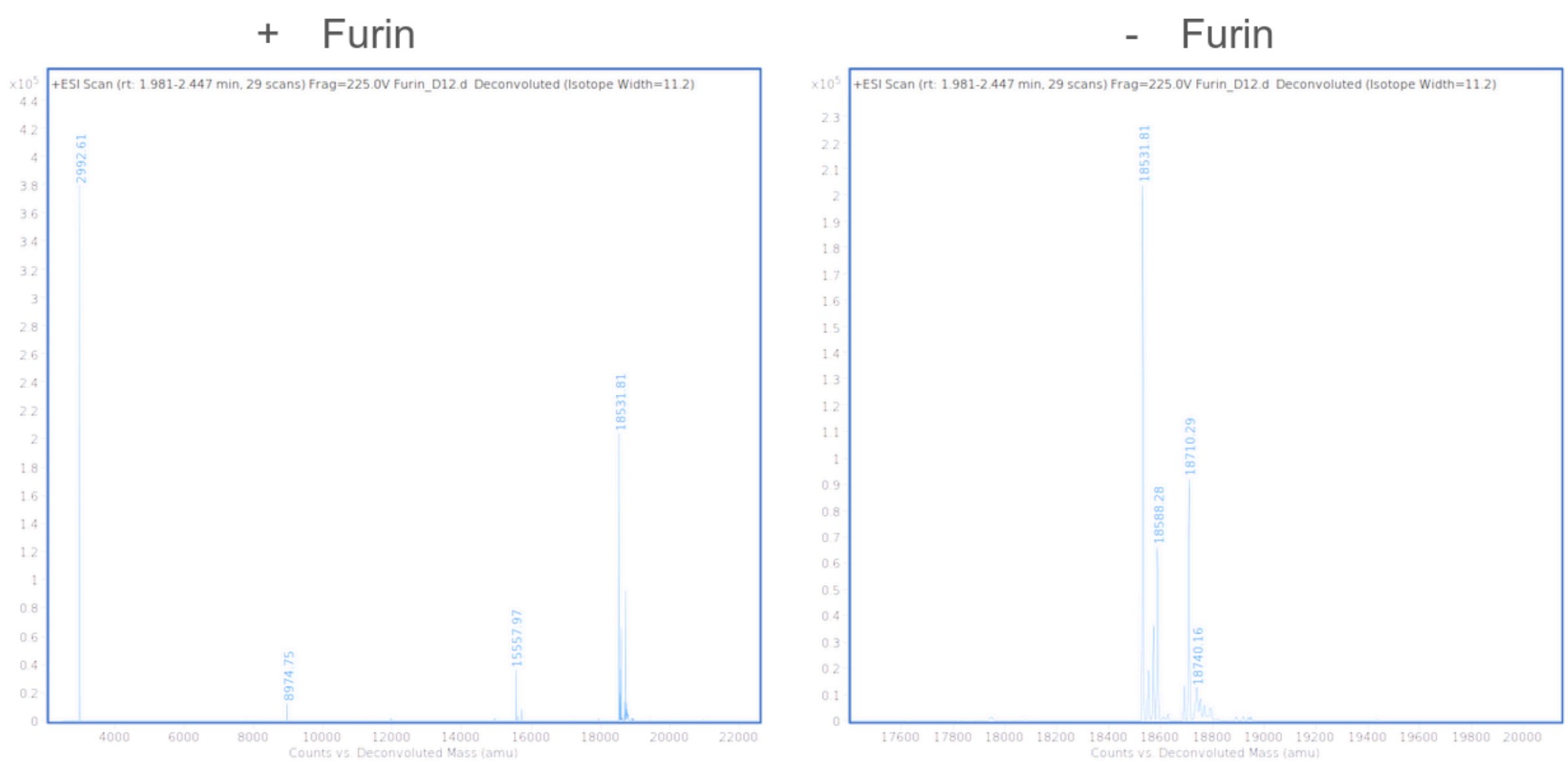

Supplementary Figure 18. Deconvolved mass spectroscopy of design D12 incubated with (left) or without (right) furin protease.

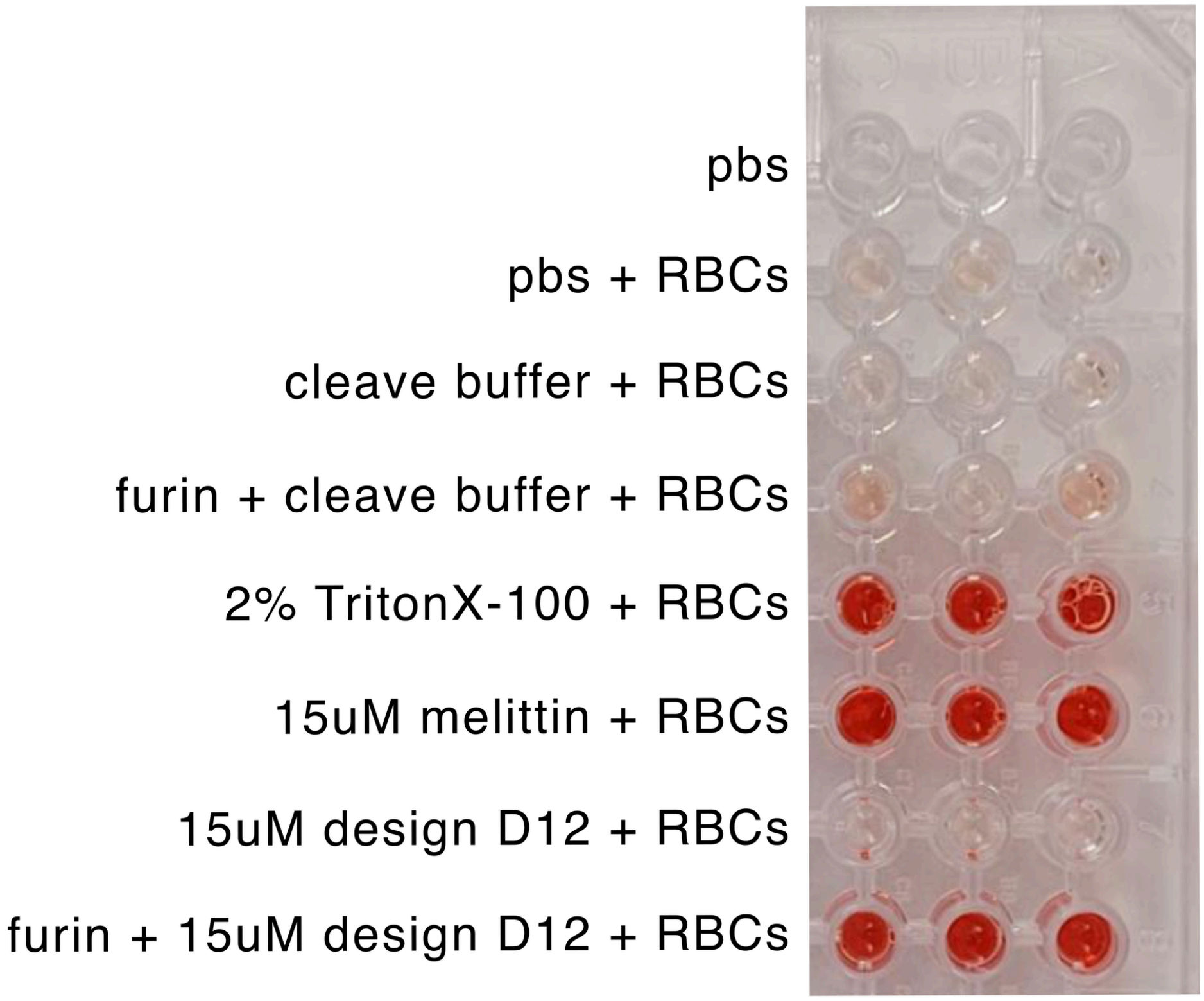

Supplementary Figure 19. Picture of red blood cell (RBC) hemolysis experiment after incubation with design D12. First four rows are negative controls PBS and cleavage buffer, followed by positive controls Triton-X-100 and purified melittin, followed by design D12 without furin protease, design D12 with furin protease.

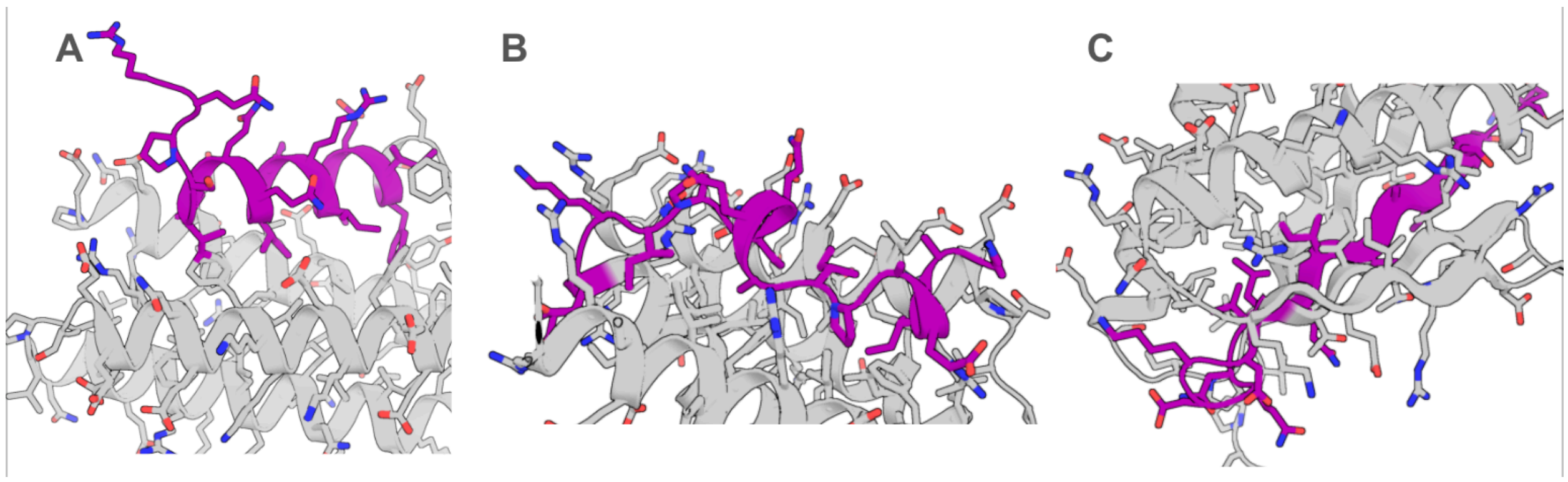

Supplementary Figure 20. Close up AF2 models of barcoded designs (A-C), with barcode side chains predicted to make hydrophobic and polar interactions with their respective scaffolds.

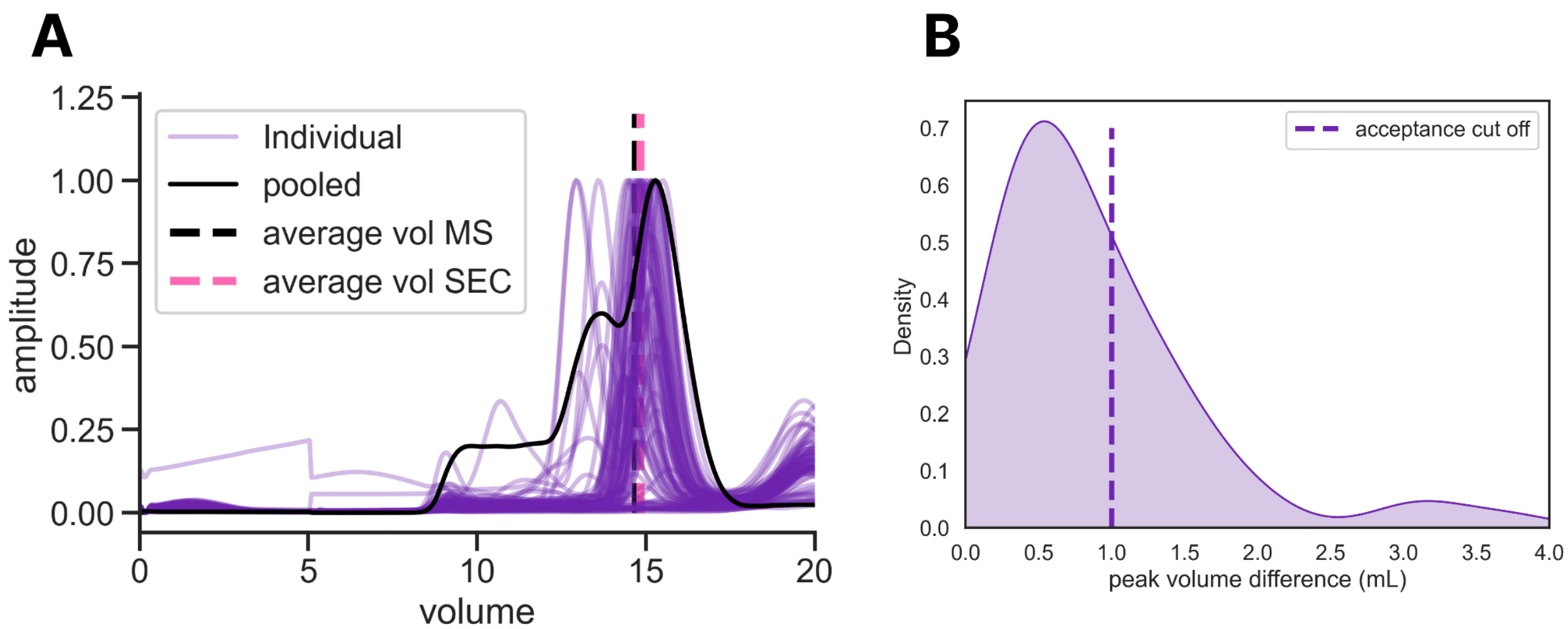

Supplementary Figure 21. (A) Overlay of SEC traces from individual (purple) and pooled (black solid) expression for barcoded designs. Dotted lines indicate that designs on average are monodisperse (pink dotted) and mass spec reconstruction (black dotted) are in agreement. (B) Peak elution volume distribution of individual SEC runs and mass spec reconstruction from pooled designs. We observed no significant difference between mass spec reconstruction and SEC peak elution volume ($p = 0.21$, two-sided Mann-Whitney U test, $n = 73$).

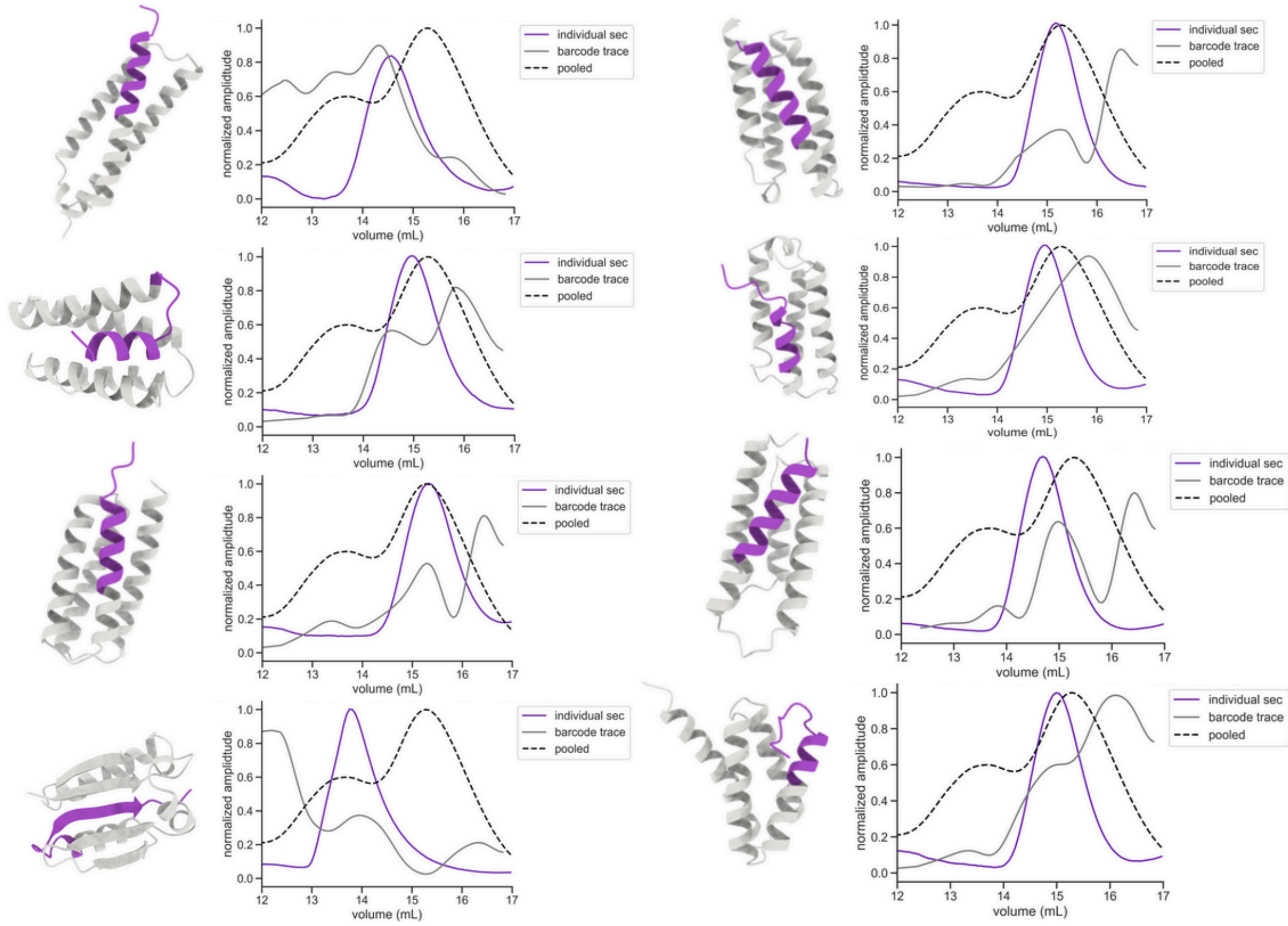

Supplementary Figure 22. Barcoded design AF2 models and corresponding SEC traces when individually expressed (gray), pooled expression followed by MS analysis (purple), pooled library (black).

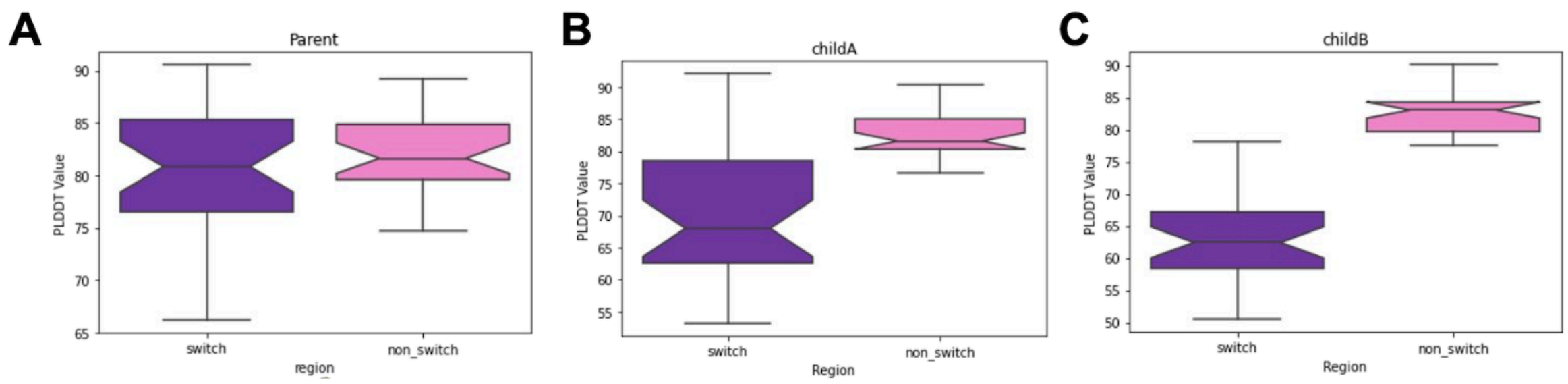

Supplementary Figure 23. Comparison of AF2 pLDDT among designed multistate proteins. Intended fold-switching region (purple) and non-fold-switching (pink) regions. All box plots boundaries indicate upper and lower quartiles, whiskers indicate the nearest quartile + 1.5*inter quartile range, center line is median. Each box plot represents *n* = 100 designs with AF2 pLDDT > 85 and RMSD to design < 2.5 Å. (A) parent design, (B) child A designs, (C) child B designs.

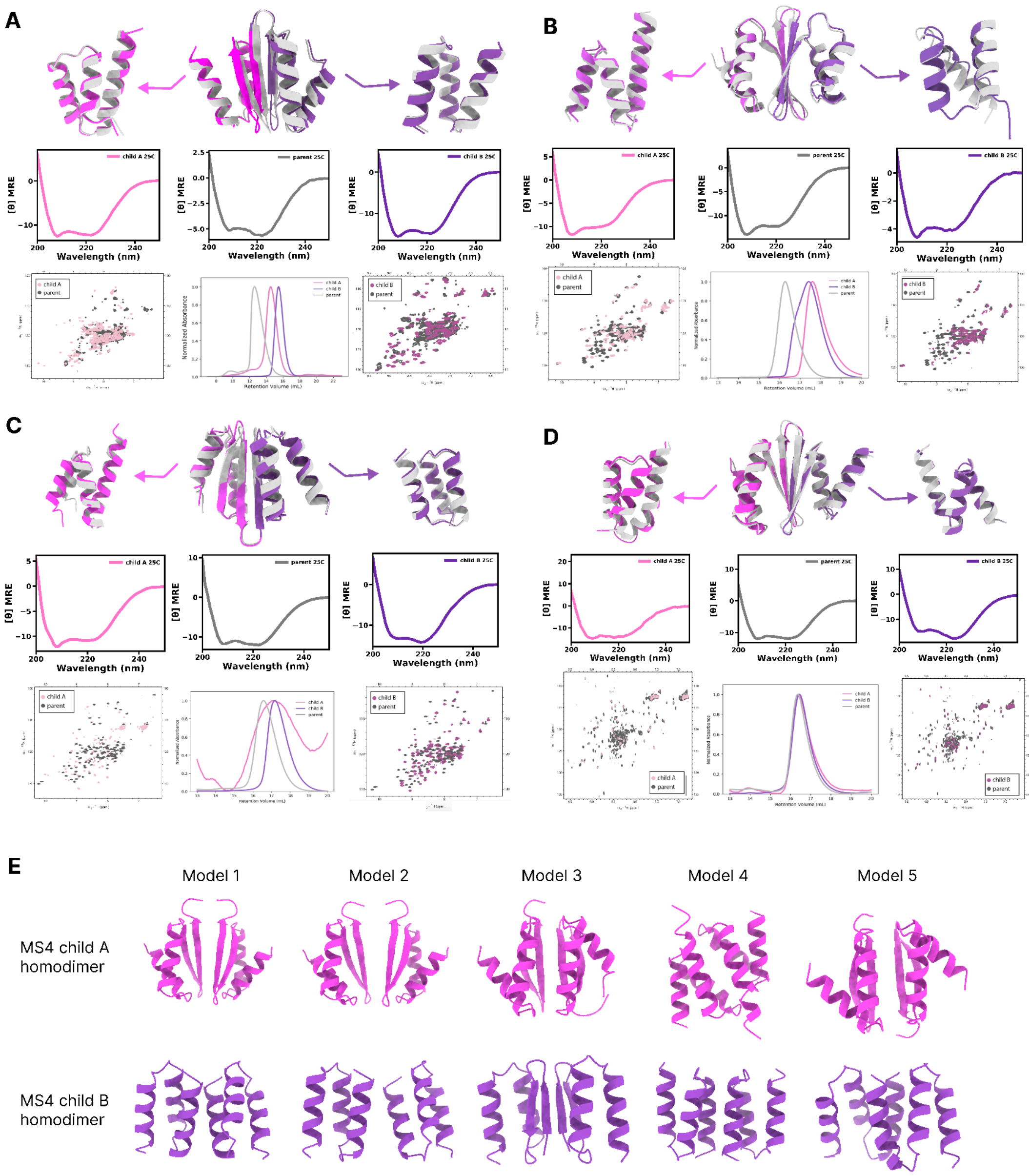

Supplementary Figure 24. Computational models and experimental characterization of multistate sequence families MS1-4. (A): MS1. (B): MS2. (C): MS3. (D): MS4. For each family of designs, top row: AF2 models (color) are in close agreement with characterized multistate design models (gray). Middle row: Circular dichroism of multistate families MS1-5. Bottom row, left and right: HSQC spectra of child A/B overlaid with parent. Bottom row, middle: overlaid size exclusion chromatography for parent, child A, and child B on a Superdex S75 10/300 Increase GL. (E) AF2 models of MS4 child A and child B homodimers predict both secondary structure states.

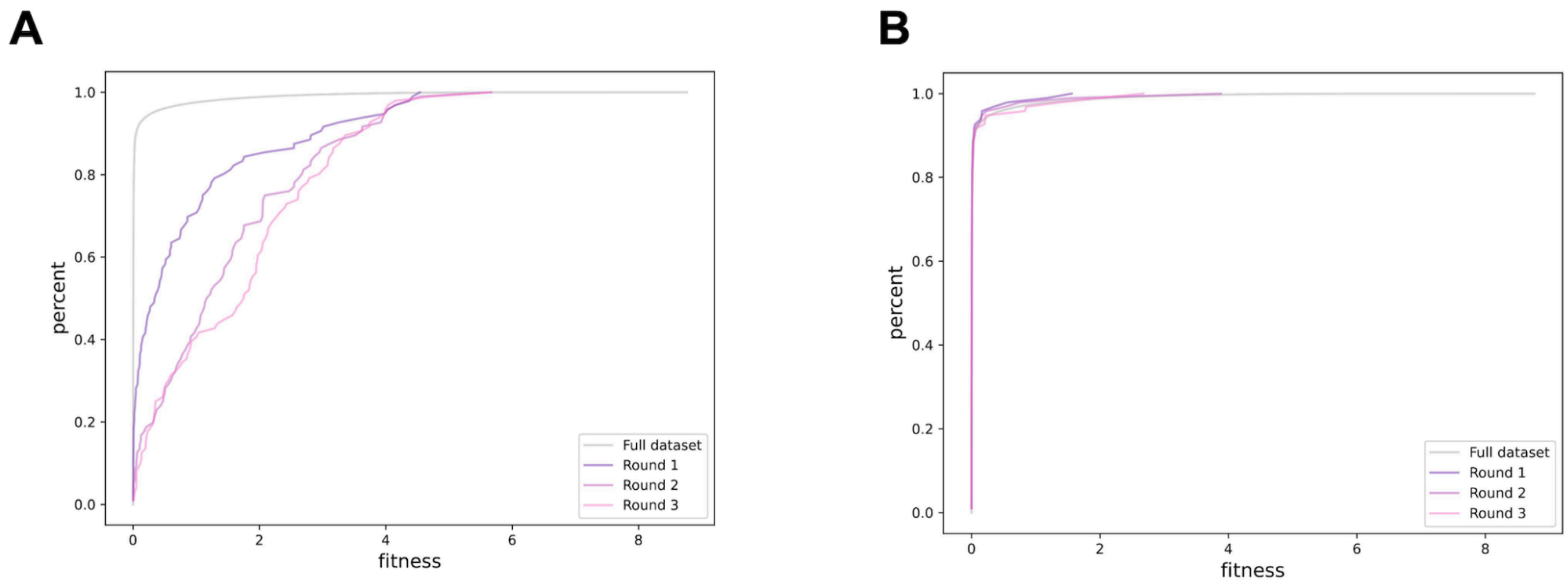

Supplementary Figure 25. Empirical cumulative distribution function plots for (A) ProteinGenerator and (B) bayesian optimization with upper confidence bound acquisition function (*n* = 96 designs per round). The full dataset shown in gray has a left shifted curve representative of a small number of variants with activity greater than 0 in the library. As curves are shifted more to the right the population of designs tends toward higher activities. The midpoint of the y-axis (0.5) represents mean fitness of the libraries.

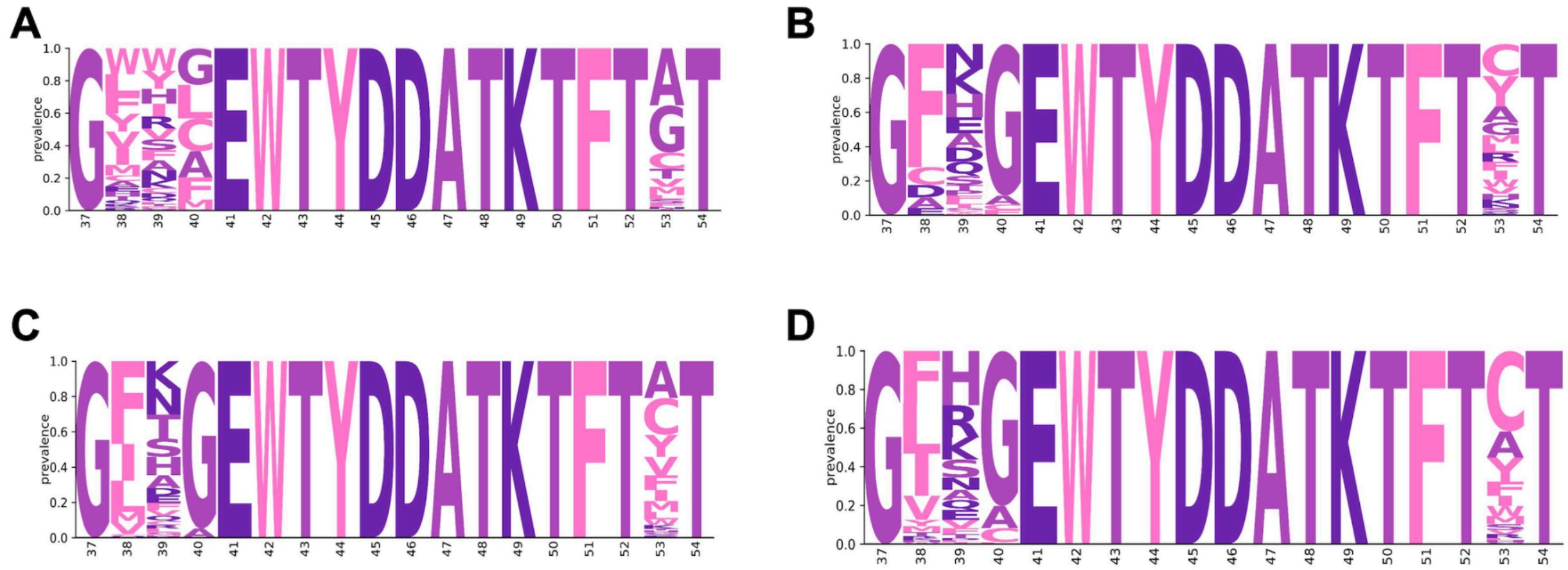

Supplementary Figure 26. Sequence logo plots of designs (A) in top 1% of full mutational landscape of GB1 and those sampled by ProteinGenerator with iterative guidance for round 1 (B), round 2 (C), and round 3 (D) (*n* = 96 designs per round).

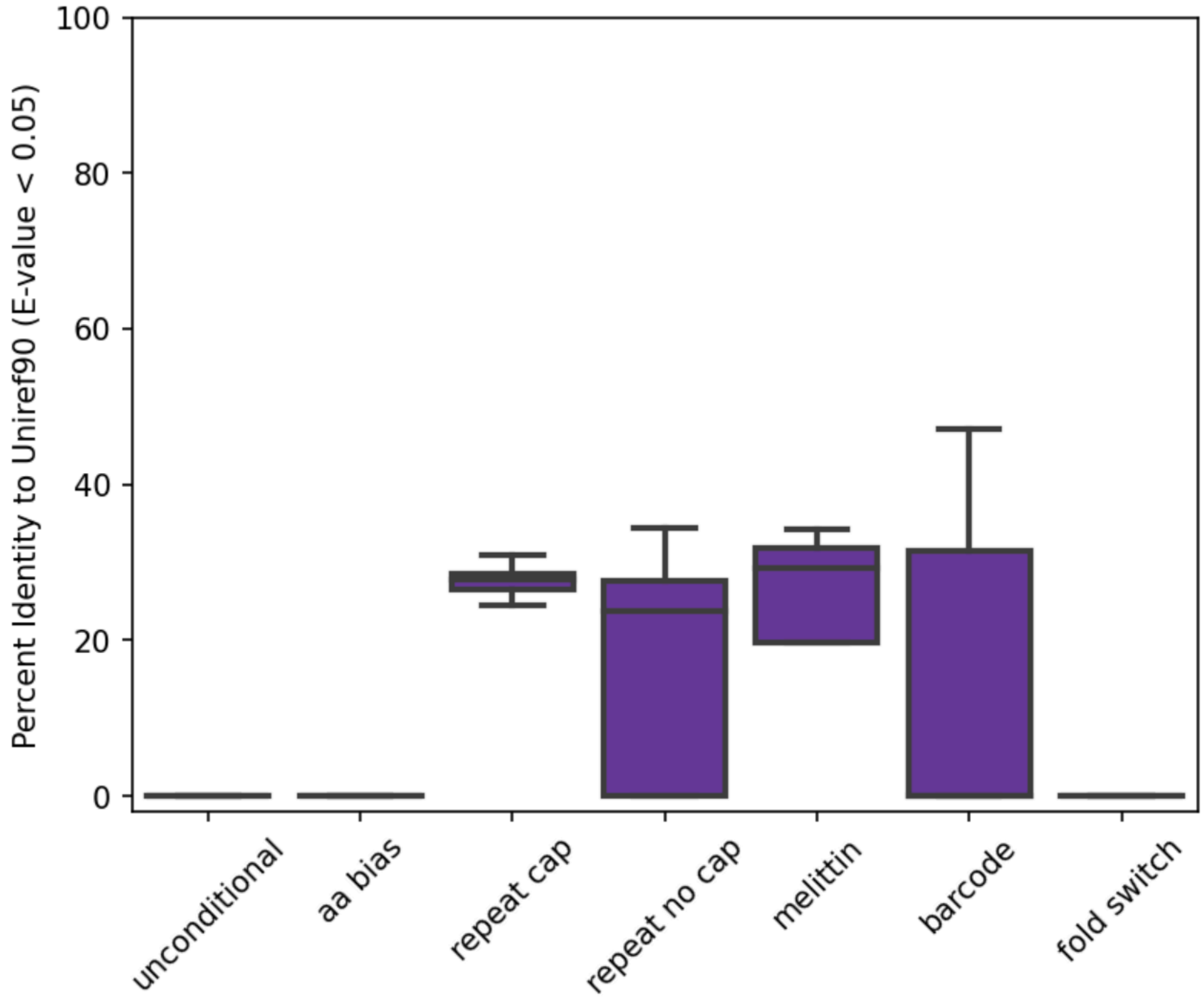

Supplementary Figure 27. Percent identity of all experimentally tested designs to Uniref90 based on design task (*n = 33* designs for unconditional, 61 for aa bias, 74 for repeat_cap, 50 for repeat no cap, 60 for melittin, 84 for barcode, and 123 for foldswitch). All box plots boundaries indicate upper and lower quartiles, whiskers indicate the nearest quartile + 1.5*inter quartile range, center line is median.

Supplementary Algorithms

Variables

1. seq : Discrete protein sequence
2. xyz : Protein Structure (3D xyz and pair features)
3. $\mathbf{x}_t$: Continuous sequence representation in logit space noised to step t
4. $\mathbf{x}_{0_{\text{selfcond}}}$: Predicted $\mathbf{x}_0$ used for self-conditioning
5. $\mathbf{x}_{t_c}$: Crop of continuous sequence input for conditioning state c
6. c : Conditioning information (str and/or seq)
7. C : Set of conditioning information (str and/or seq)
8. T : Number of diffusion steps
9. t : Diffusion timestep
10. $G : R^{L\times 20} \rightarrow R$ guidance function
11. β_t : Variance schedule
12. $\bar{\beta}_t : \prod_{s=1}^{t} \beta_s$
13. $\alpha_t : 1 - \beta_t$
14. $\bar{\alpha}_t : \prod_{s=1}^{t} \alpha_s$
15. L : Length (Number of Amino Acids)
16. λ : Guidance scale
17. γ_j : Scaling factor for state j
18. p : Fraction of sequence to apply guidance
19. idx_{aa} : Amino acid index
20. idx_{bias} : Indices of residues to apply guidance

Algorithm 1 Training

function $\text{TrainStep}(seq, xyz, c)$

  $\mathbf{x}_0 \leftarrow \text{Onehot}(seq) * 2 - 1$ ▷ convert sequence to [-1,1]

  $t \sim \text{Uniform}(\{1, \dots, T\})$

  $\epsilon \sim \mathcal{N}(0, I)$

  $\mathbf{x}_t \leftarrow \sqrt{\bar{\alpha}_t}\mathbf{x}_0 + \sqrt{1 - \bar{\alpha}_t}\epsilon$

  $\mathbf{x}_{t+1} \leftarrow \sqrt{\bar{\alpha}_{t+1}}\mathbf{x}_0 + \sqrt{1 - \bar{\alpha}_{t+1}}\epsilon$

  $\mathbf{x}_{0_{\text{selfcond}}}, xyz_{pred} \leftarrow \text{stopgrad}(\text{model}(\mathbf{x}_{t+1}, t+1, c, \mathbf{x}_{0_{\text{selfcond}}}))$

  $\mathbf{x}_{0_{\text{pred}}}, xyz_{pred} \leftarrow \text{model}(\mathbf{x}_t, t, c, \mathbf{x}_{0_{\text{selfcond}}})$

  $\text{cce\_loss} \leftarrow \text{CCE\_loss}(\mathbf{x}_0, \mathbf{x}_{0_{\text{pred}}})$

  $\text{kl\_loss} \leftarrow \text{KL\_reconstruction\_loss}(\mathbf{x}_0, \mathbf{x}_{0_{\text{pred}}}, t)$

  $\text{str\_loss} \leftarrow \text{Structure\_loss}(xyz, xyz_{\text{pred}})$

  Take gradient descent step on $\text{cce\_loss} + \text{kl\_loss} + \text{str\_loss}$

end function

function $\text{KL\_RECONSTRUCTION\_LOSS}(\mathbf{x}_0, \mathbf{x}_{0_{\text{pred}}}, t, \beta_t, \beta_{t-1}, \alpha_t, \bar{\alpha}_t, \bar{\alpha_{t-1}})$

  $\mathbf{x}_{t-1_{\text{pred}}} \leftarrow \frac{\sqrt{\bar{\alpha}_{t-1}}\beta_t}{1-\bar{\alpha}_t}\mathbf{x}_{0_{\text{pred}}} + \frac{\sqrt{\alpha_t}(1-\bar{\alpha}_{t-1})}{1-\bar{\alpha}_t}\mathbf{x}_t$

  $\mathbf{x}_{t-1} \leftarrow \frac{\sqrt{\bar{\alpha}_{t-1}}\beta_t}{1-\bar{\alpha}_t}\mathbf{x}_0 + \frac{\sqrt{\alpha_t}(1-\bar{\alpha}_{t-1})}{1-\bar{\alpha}_t}\mathbf{x}_t$

  $\sigma_t^2 \leftarrow \frac{1-\bar{\alpha}_{t-1}}{1-\bar{\alpha}_t}\beta_t$

  return $\text{KL}(\mathbf{x}_{t-1}, \mathbf{x}_{t-1_{\text{pred}}}, \sigma_t^2)$

end function

Algorithm S1. ProteinGenerator finetuning procedure.

Algorithm 2 Inference

function SAMPLE(T, L, λ,c,G)
  $\mathbf{x}_t \sim \mathcal{N}(0, I)_{L,20}$
  $\mathbf{x}_{0_{\text{selfcond}}} \leftarrow$ None
  for i in range(T) **do**
    $t \leftarrow T - i$
    $\mathbf{x}_0, xyz \leftarrow \text{model}(\mathbf{x}_t, t, c, \mathbf{x}_{0_{\text{selfcond}}})$
    $\mathbf{g} \leftarrow \nabla_{\mathbf{x}} G(\mathbf{x}_0, t)$
    $\mathbf{x}_t \leftarrow \sqrt{\bar{\alpha}_{t-1}}(\mathbf{x}_0 + \lambda \mathbf{g}) + \sqrt{1 - \bar{\alpha}_{t-1}}\epsilon$
    $\mathbf{x}_{0_{\text{selfcond}}} \leftarrow \mathbf{x}_0$
  end for
  $seq \leftarrow \text{argmax}(\mathbf{x}_0)$
  return seq, xyz
end function

Algorithm S2. ProteinGenerator inference procedure.

Algorithm 3 Sequence Bias

function AMINOACIDBIAS($\mathbf{x}_{0_{\text{pred}}}, L, p, idx_{aa}$)

  $\mathbf{g} \leftarrow 0^{L \times 20}$

  $n \leftarrow \text{int}(pL)$

  $idx_{bias} \leftarrow \text{topK}(\mathbf{x}_{0_{\text{pred}}}[:, idx_{aa}], n)$

  $\mathbf{g}[idx_{bias} : idx_{aa}] \leftarrow 1$

  return g

end function

Algorithm S3. Amino acid bias procedure.

Algorithm 4 Multistate Inference

function SAMPLEMULTISTATE(T, L, λ, C, γ)
  $\mathbf{x}_t \sim \mathcal{N}(0, I)_{L,20}$
  $\mathbf{x}_{0_{\text{selfcond}}} \leftarrow$ None
  for i in range(T) **do**
    $t \leftarrow T - i$
    for j, c in enumerate(C) **do**
      $\mathbf{x}_{0_j}, xyz_j \leftarrow \text{model}(\mathbf{x}_{t_c}, t, c, \mathbf{x}_{0_{\text{selfcond}}})$
    end for
    $\mathbf{x}_0 \leftarrow \sum_{j=0}^{|C|} \gamma_j \mathbf{x}_{0_j}$
    $\mathbf{x}_t \leftarrow \sqrt{\bar{\alpha}_{t-1}}\mathbf{x}_0 + \sqrt{1 - \bar{\alpha}_{t-1}}\epsilon$
    $\mathbf{x}_{0_{\text{selfcond}}} \leftarrow \mathbf{x}_0$
  end for
  $seq \leftarrow \text{argmax}(\mathbf{x}_0)$
  return seq, xyz
end function

Algorithm S4. Multistate inference procedure.

Supplementary Tables

Task	Timesteps	Criteria	Passing rate	Conditioning Information
Unconditional	25	pLDDT > 90 RMSD < 2	6%	None
Compositional bias	25	pLDDT > 85 RMSD < 2	7%	AA composition
Repeat cap	50	pLDDT > 80 RMSD < 2	2.3%	DSSP
Repeat no cap	50	pLDDT > 80 RMSD < 2	6%	DSSP
Melittin	25	pLDDT > 85 RMSD < 2	1.7 %	DSSP, sequence motif scaffolding
Barcoding C-term	50	pLDDT > 85 RMSD < 2	9%	Sequence motif scaffolding
Multistate	25	pLDDT > 75 RMSD < 3	0.05%	DSSP

Supplementary Table 1: Inference timesteps, filtering criteria, success rates, and conditioning information for all experimentally tested design tasks. Different timesteps and filtering criteria are determined by observed success rates in test runs, as well as design task complexity, available compute, and number of designs that can be ordered.

GPU	Time step	Length	Time (seconds)
A5000	50	50	110
A5000	50	100	111
A5000	50	200	165
A5000	50	300	316

Supplementary Table 2: PG runtime to generate one protein on an A5000 GPU in 50 timesteps.
